# Supplementary figures and images for: The TRRAP transcription cofactor represses interferon-stimulated genes in colorectal cancer cells
Source: eLife. 2022 Mar 4;11:e69705. doi: 10.7554/eLife.69705 (PMC8926402; doi:10.7554/eLife.69705)

**A**

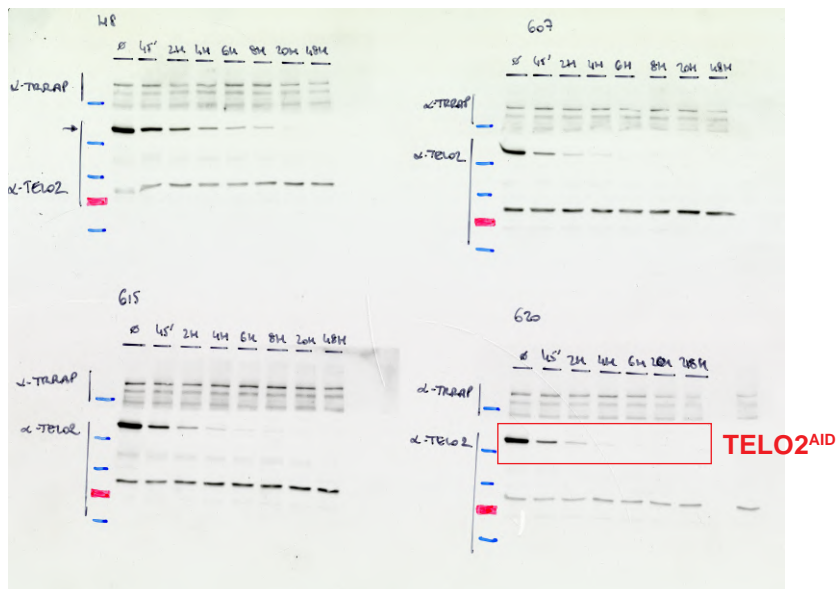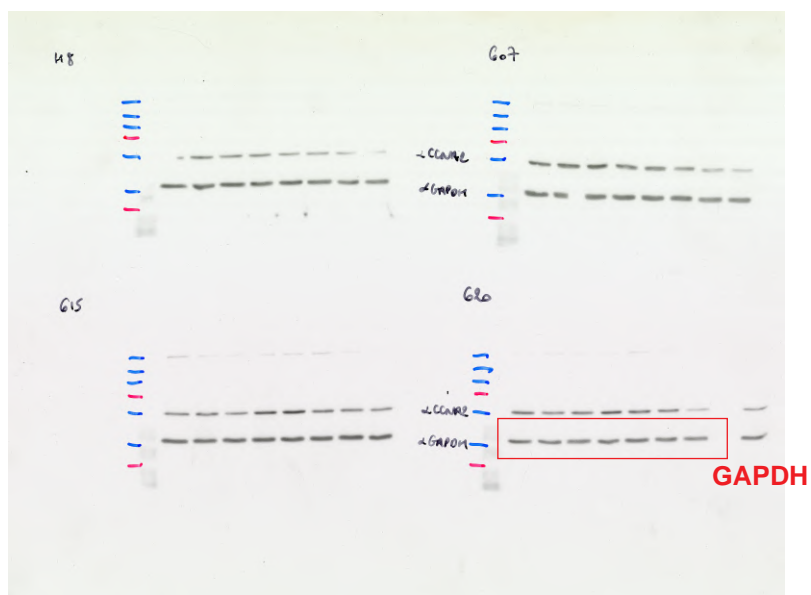

**B**

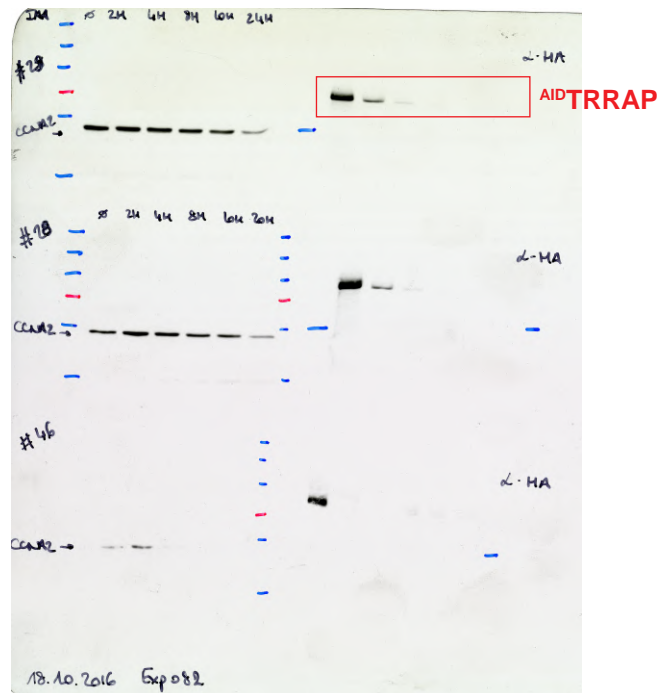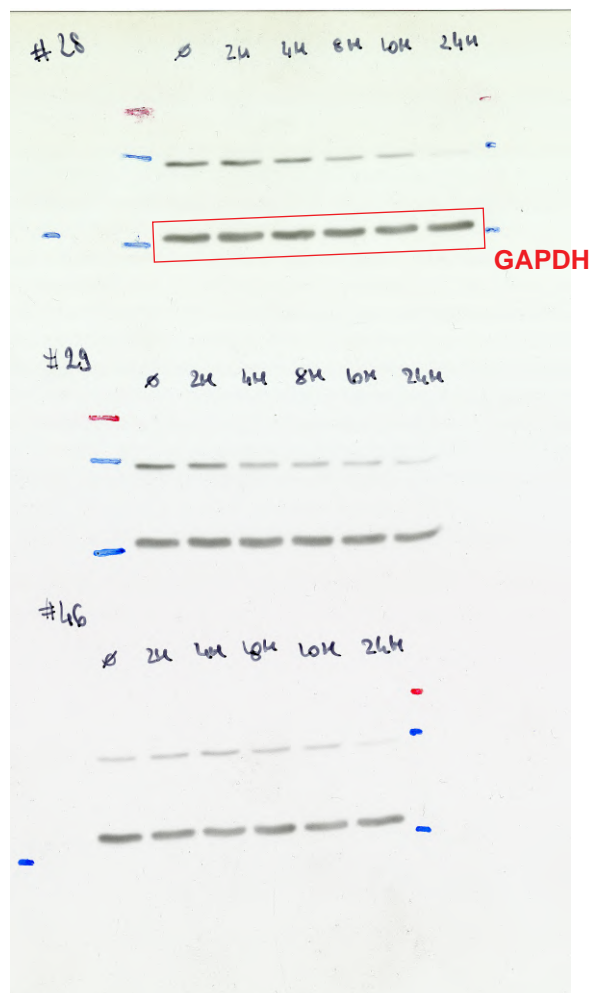

Supplement: Source data 1. [file elife-69705-data1.zip › JPEG/Figure1A-B-SourceData1_compressed.pdf]

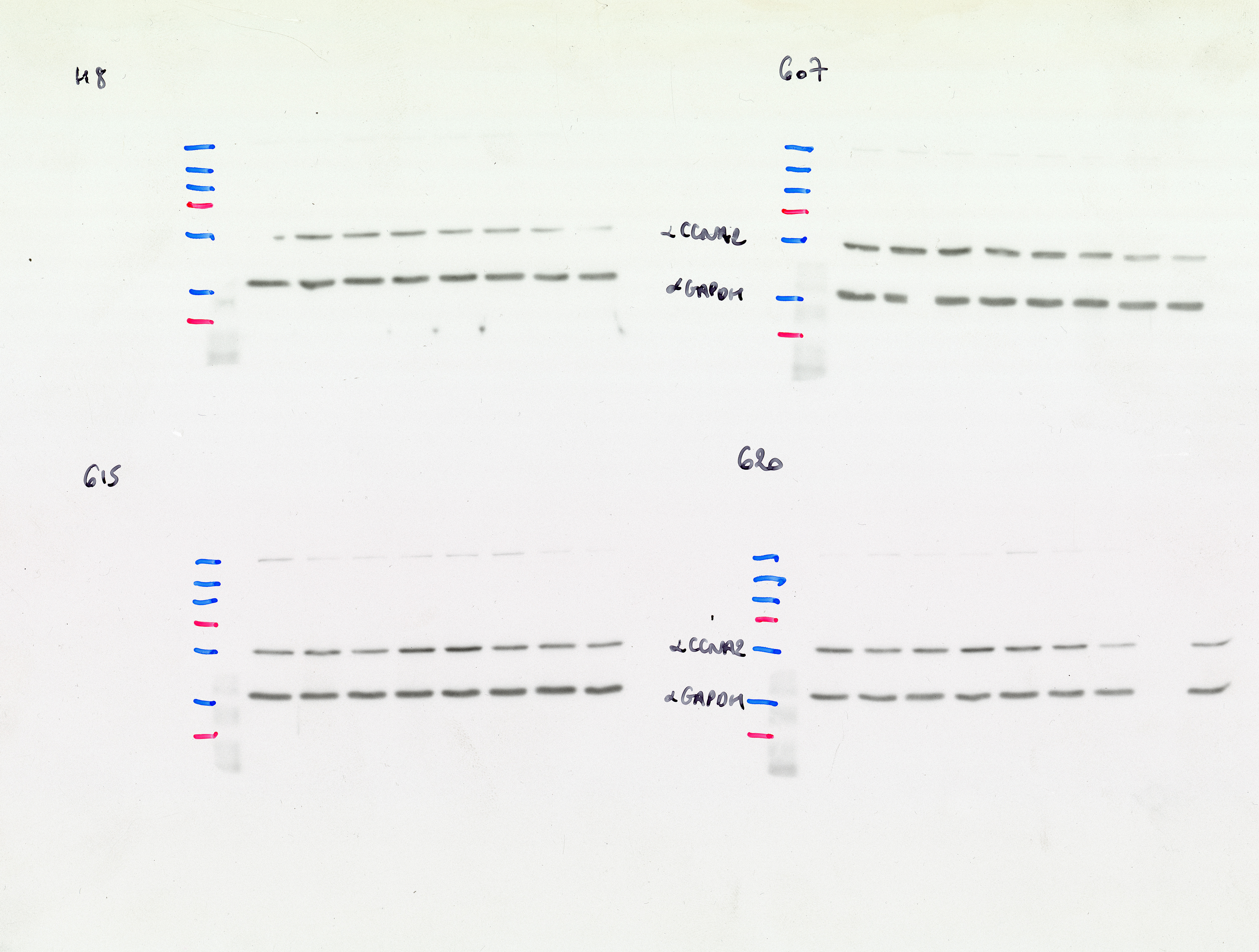

Supplement: Source data 1. [file elife-69705-data1.zip › JPEG/Figure1A-SourceData1-GAPDH.jpg]

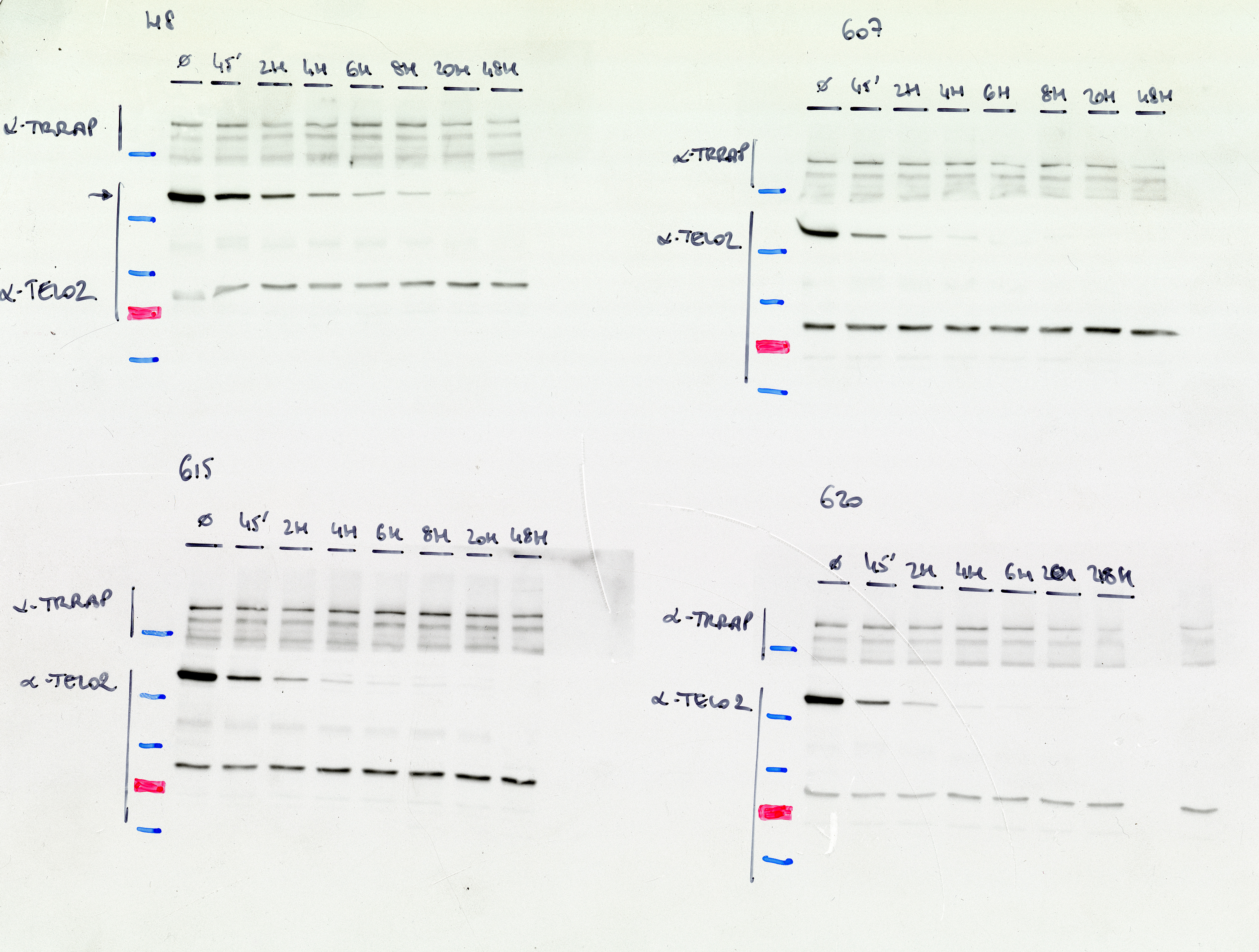

Supplement: Source data 1. [file elife-69705-data1.zip › JPEG/Figure1A-SourceData1-TELO2.jpg]

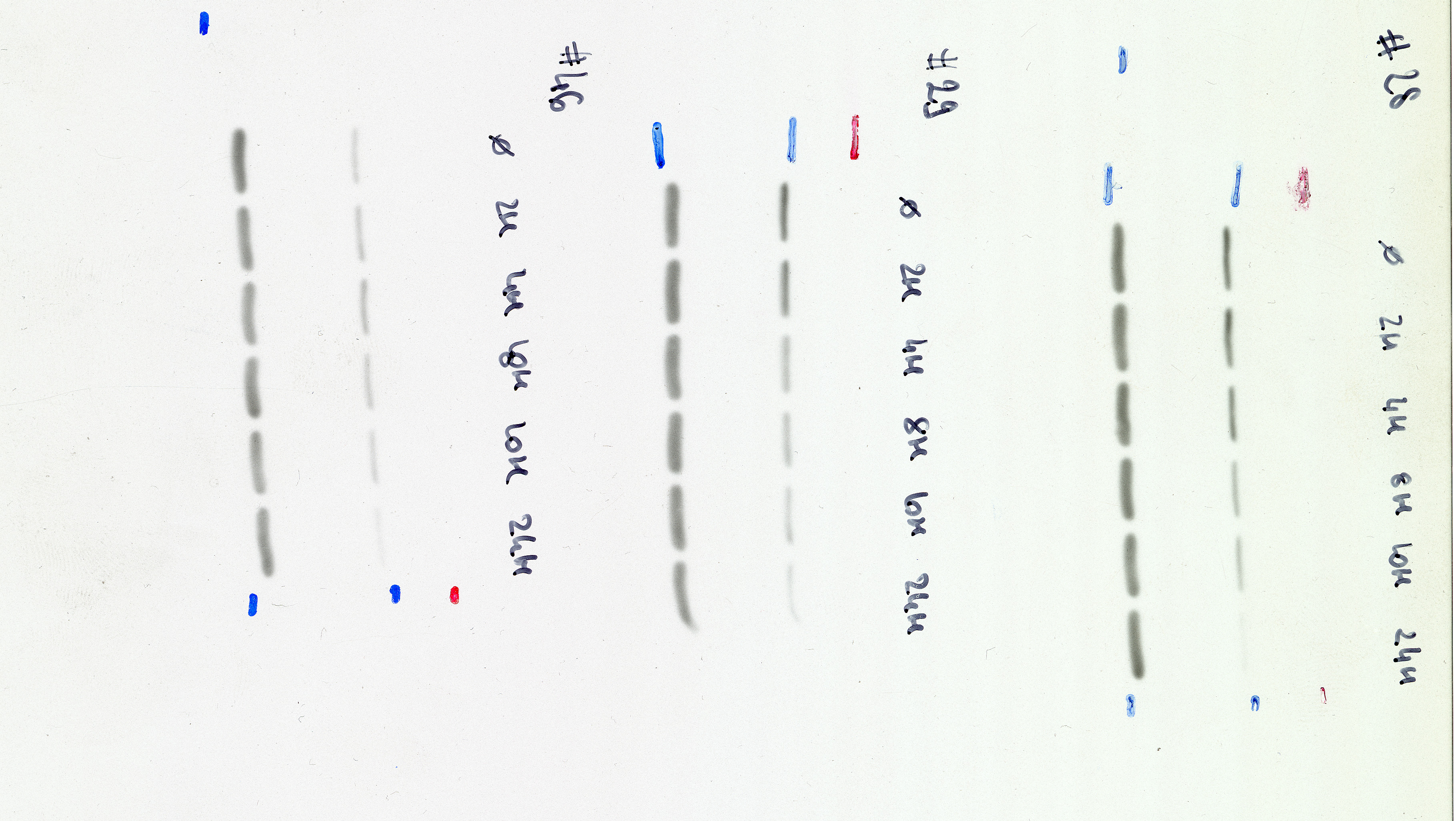

Supplement: Source data 1. [file elife-69705-data1.zip › JPEG/Figure1B-SourceData1-GAPDH.jpg]

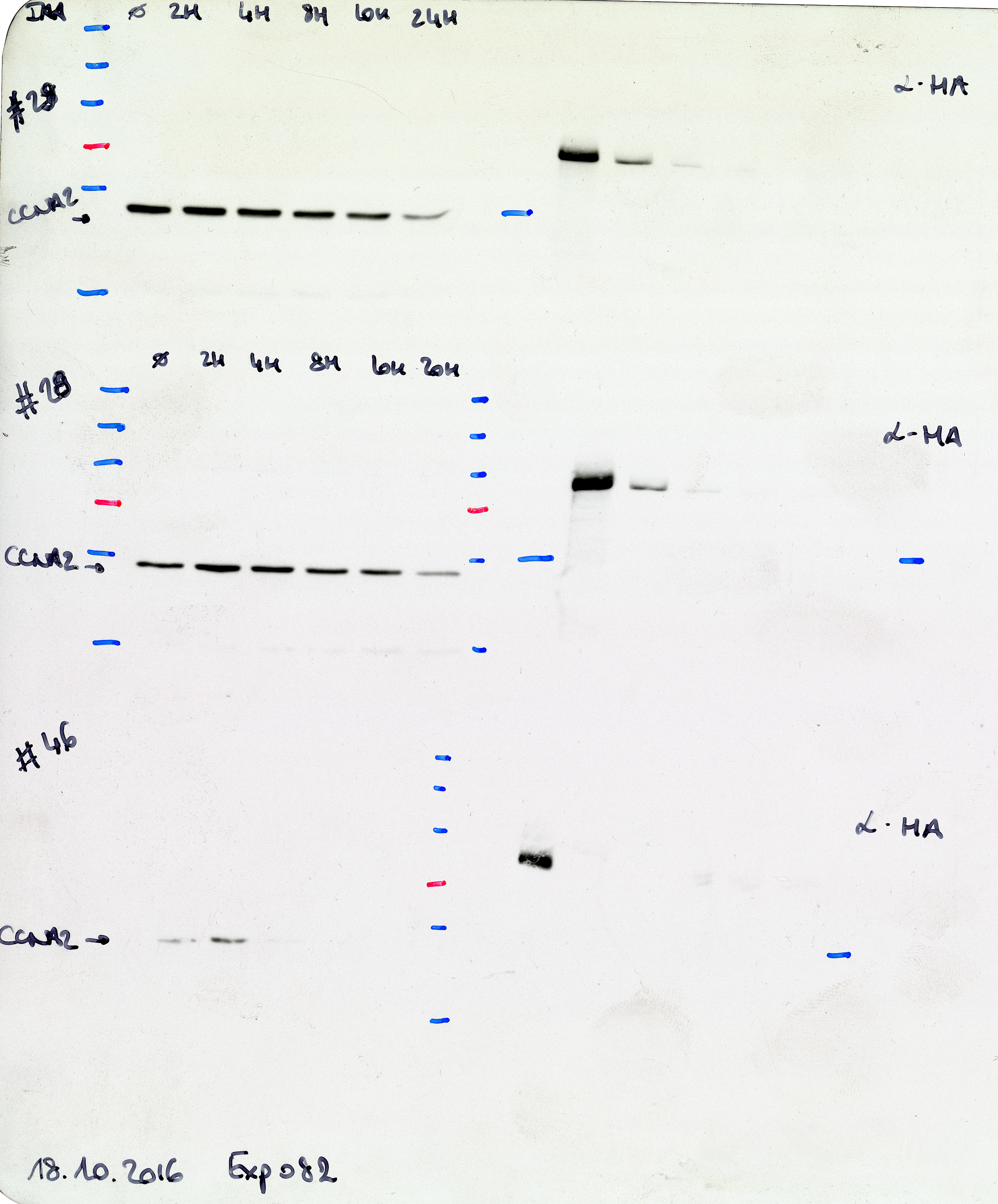

Supplement: Source data 1. [file elife-69705-data1.zip › JPEG/Figure1B-SourceData1-TRRAP.jpg]

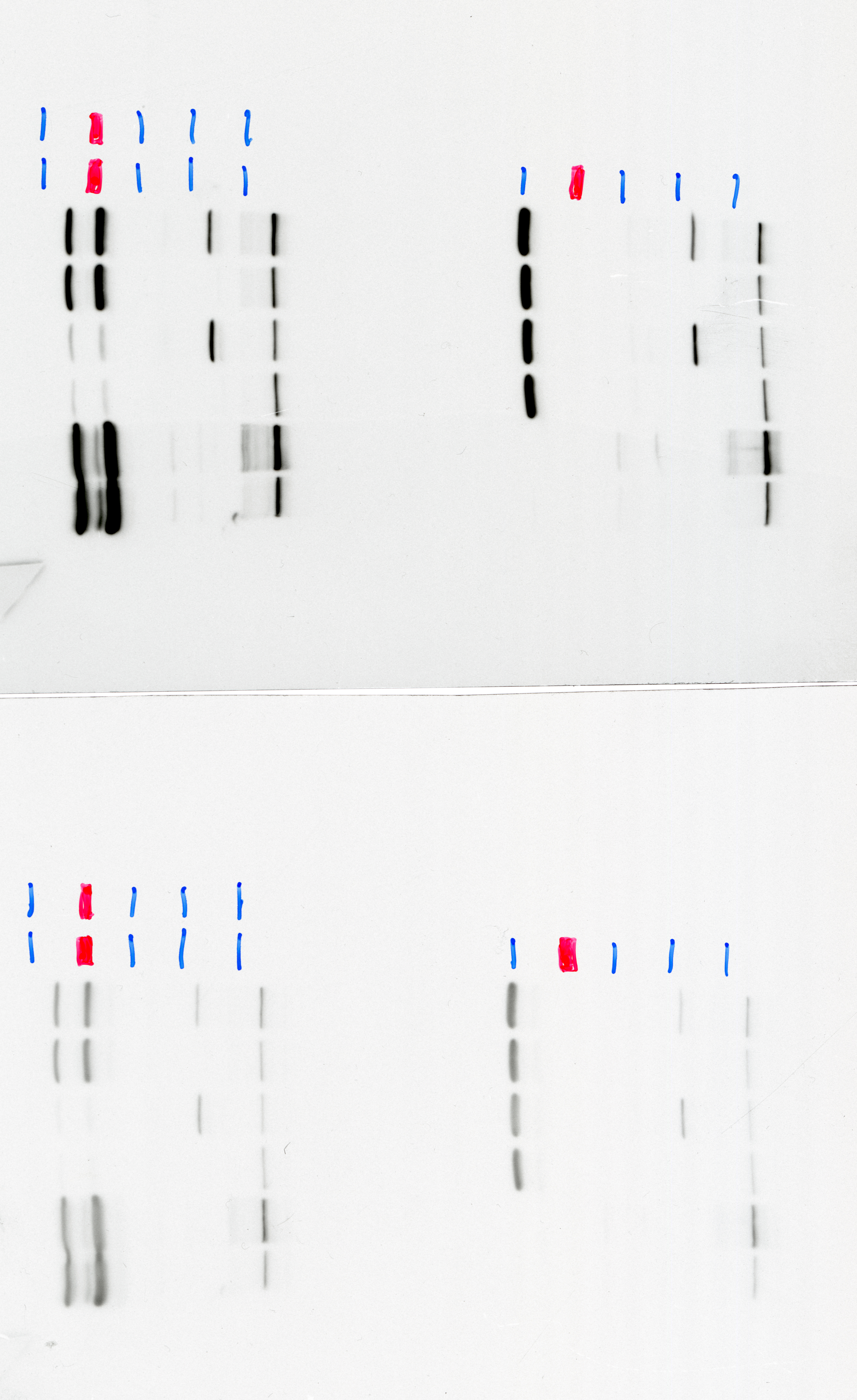

Supplement: Source data 1. [file elife-69705-data1.zip › JPEG/Figure1E-SourceData1.jpg]

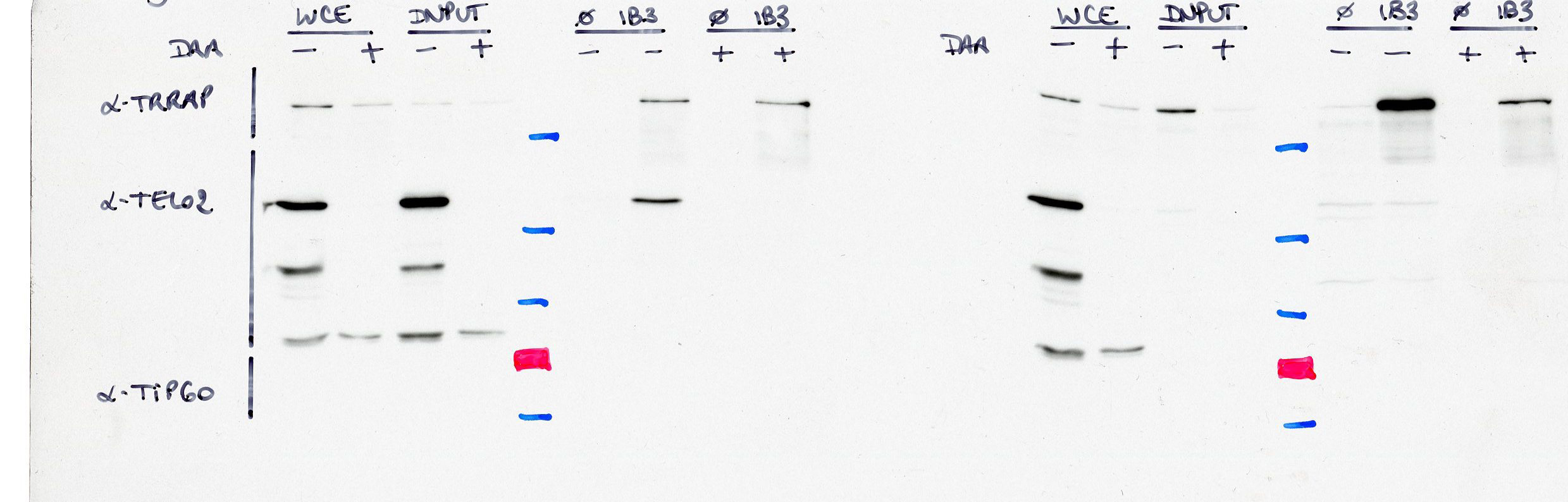

Supplement: Source data 1. [file elife-69705-data1.zip › JPEG/Figure1F-SourceData1.jpg]

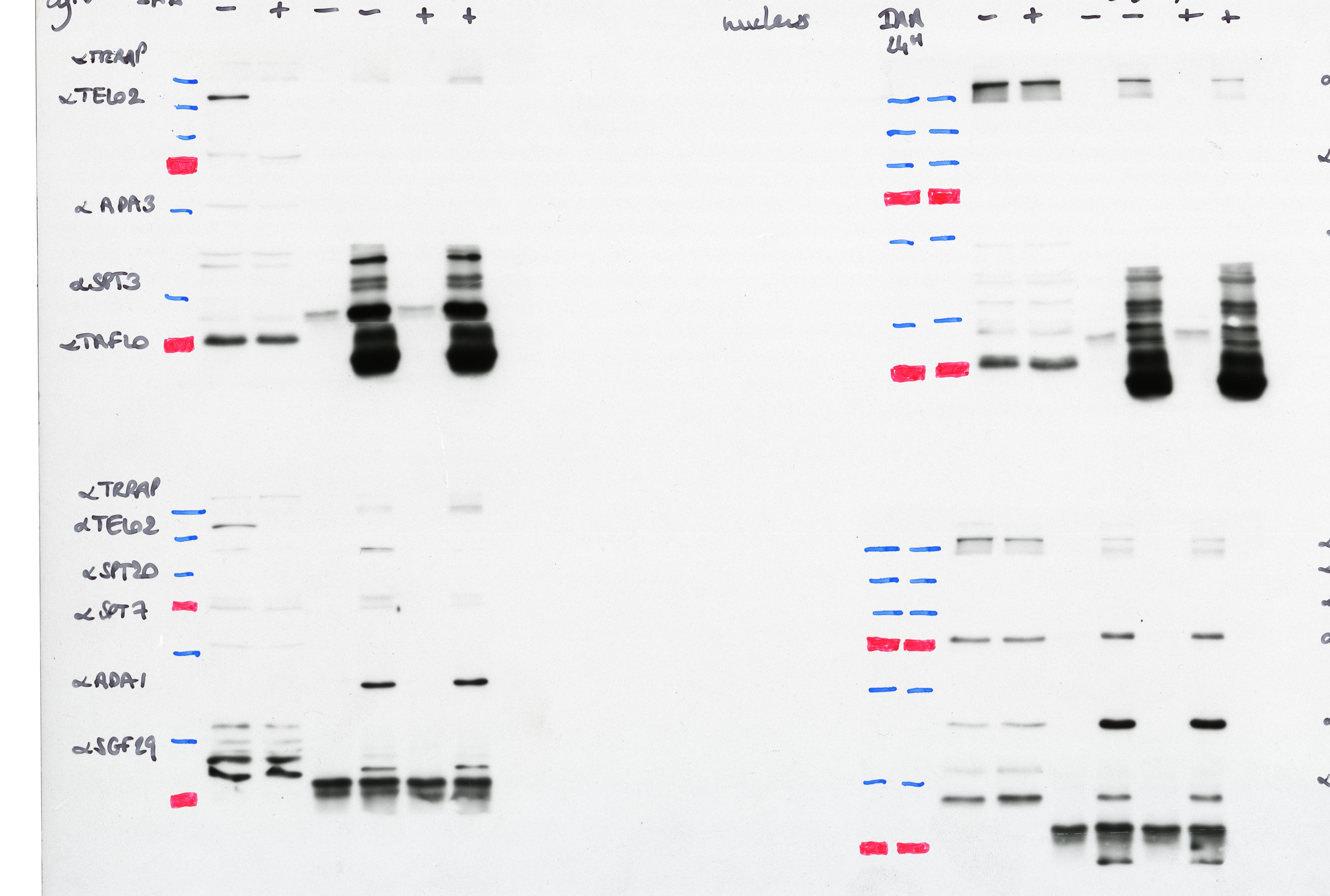

Supplement: Source data 1. [file elife-69705-data1.zip › JPEG/Figure1G-SourceData1short.jpg]

G

short exposure

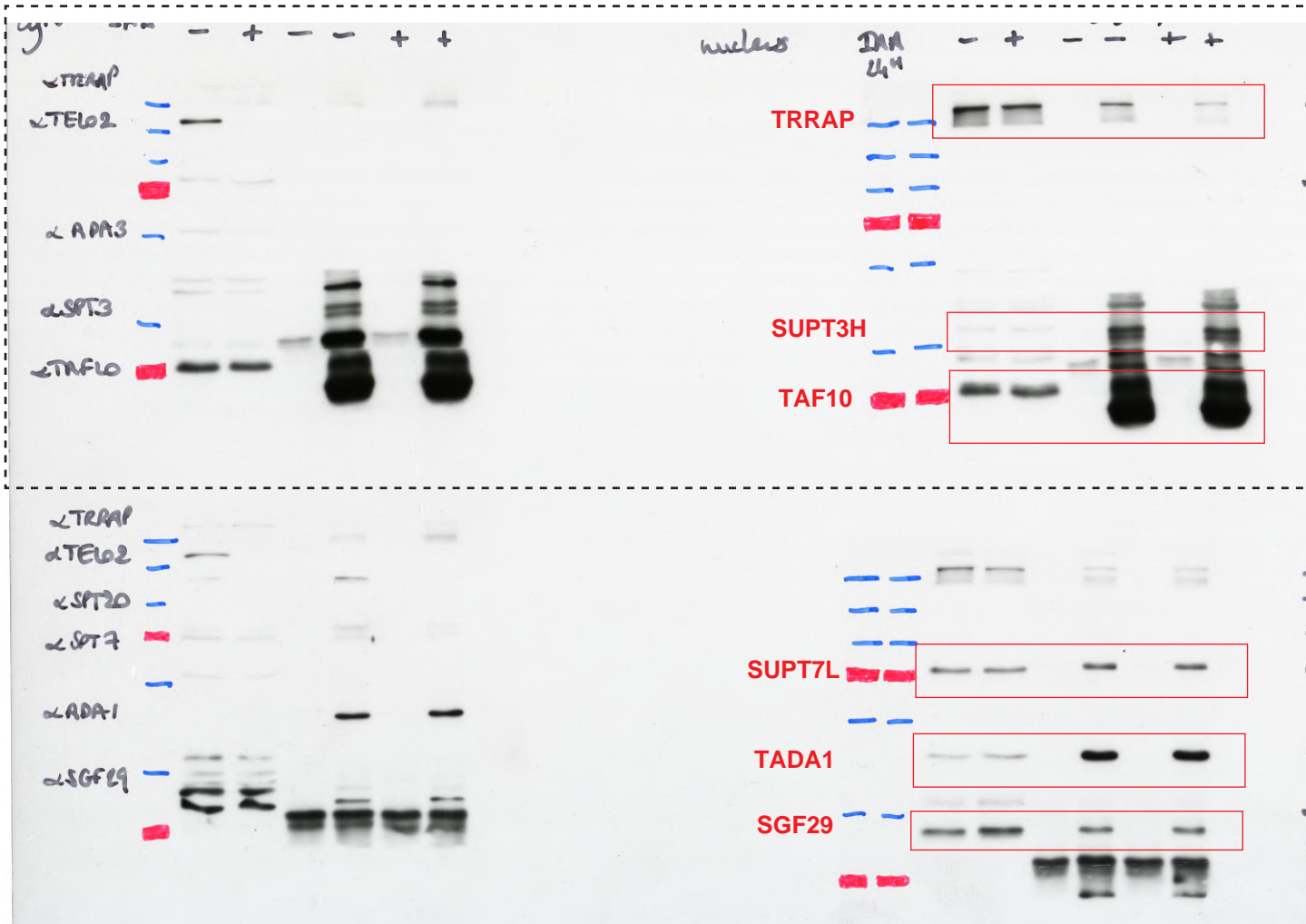

mid-exposure

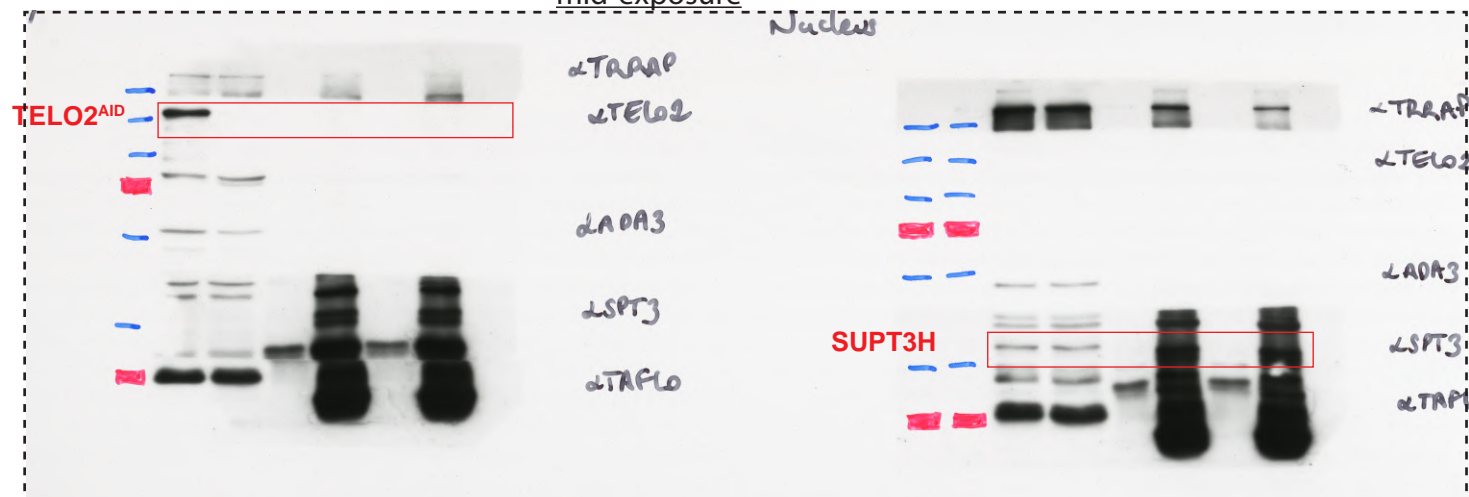

long exposure

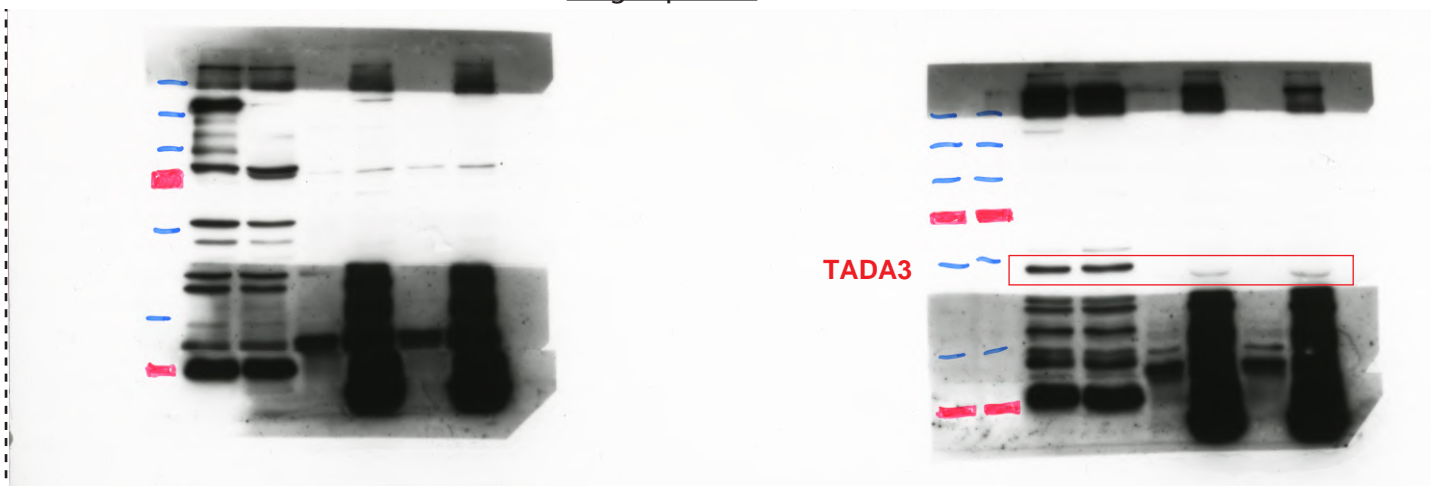

Supplement: Source data 1. [file elife-69705-data1.zip › JPEG/Figure1G-SourceData1_compressed.pdf]

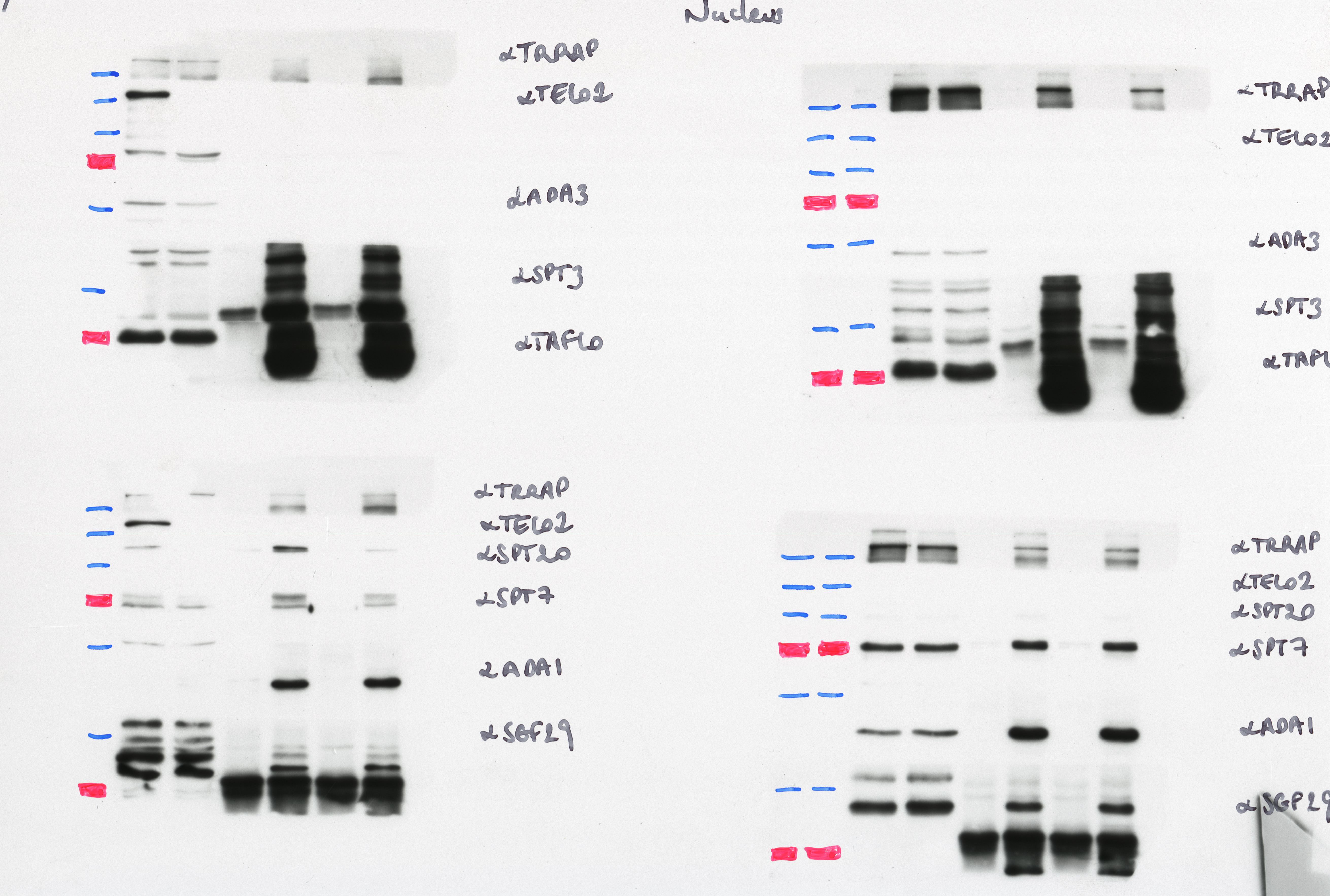

Supplement: Source data 1. [file elife-69705-data1.zip › JPEG/Figure1G-SourceData2mid.jpg]

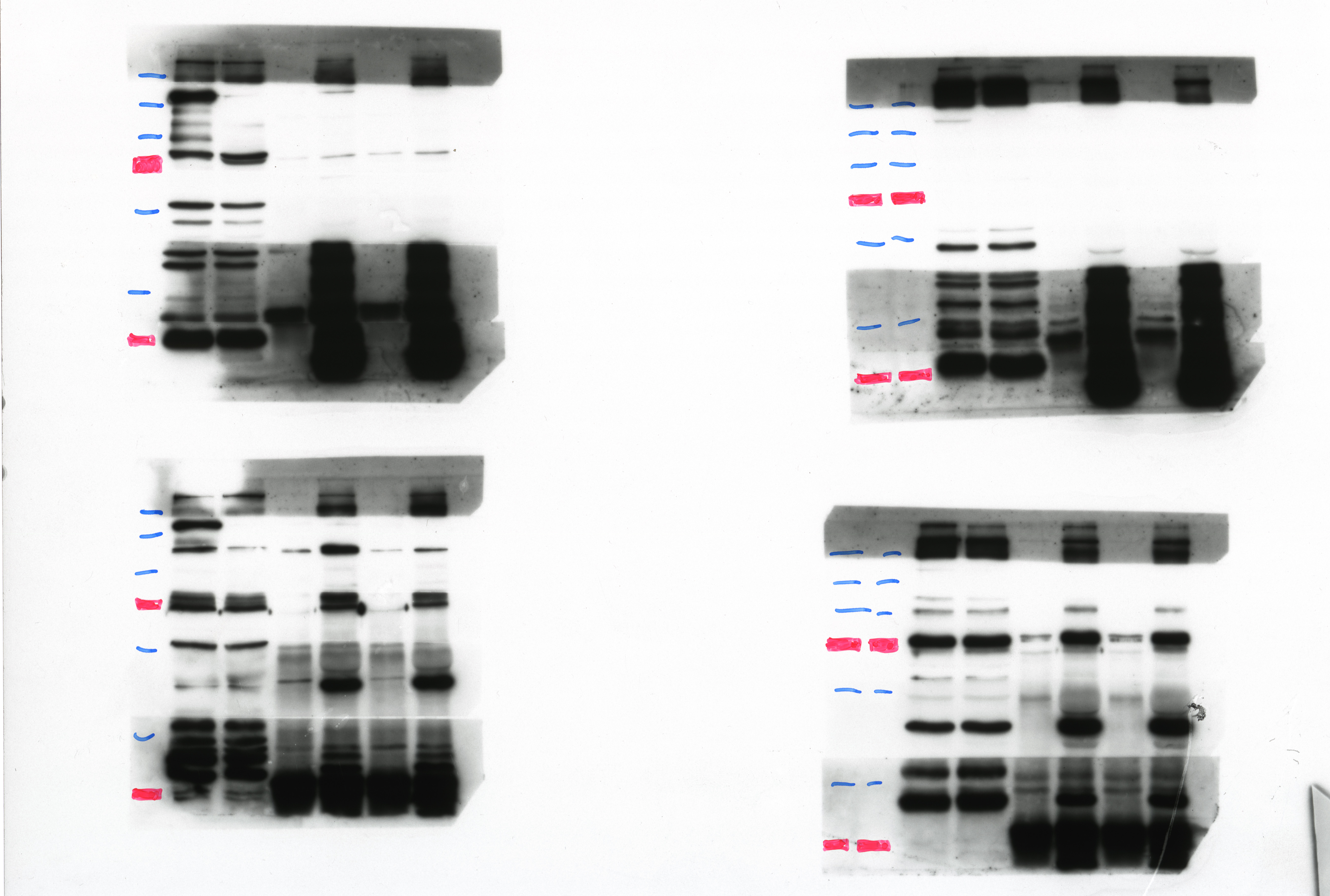

Supplement: Source data 1. [file elife-69705-data1.zip › JPEG/Figure1G-SourceData3long.jpg]

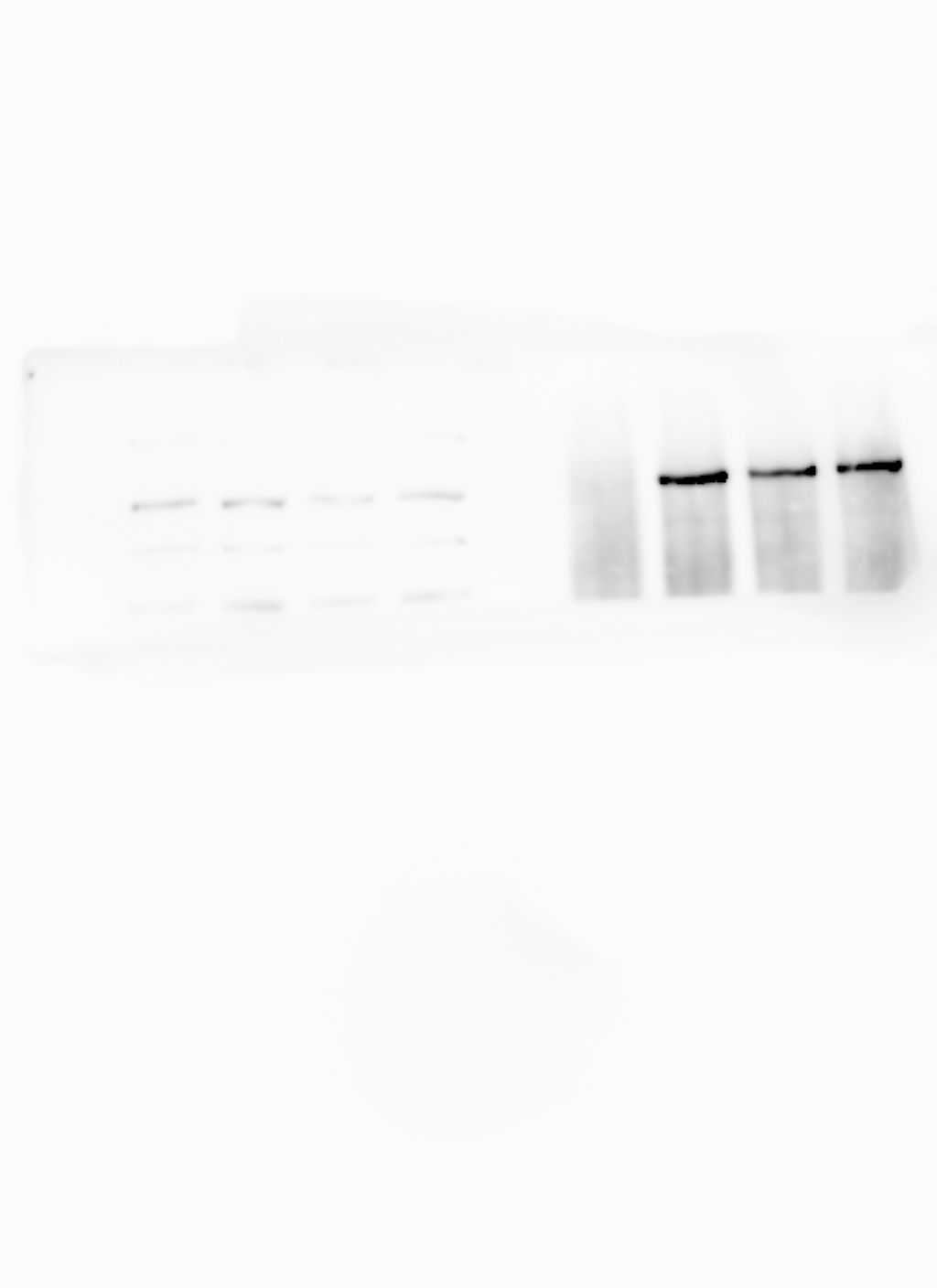

Supplement: Source data 1. [file elife-69705-data1.zip › JPEG/Figure1H-SourceData1-EP400.jpg]

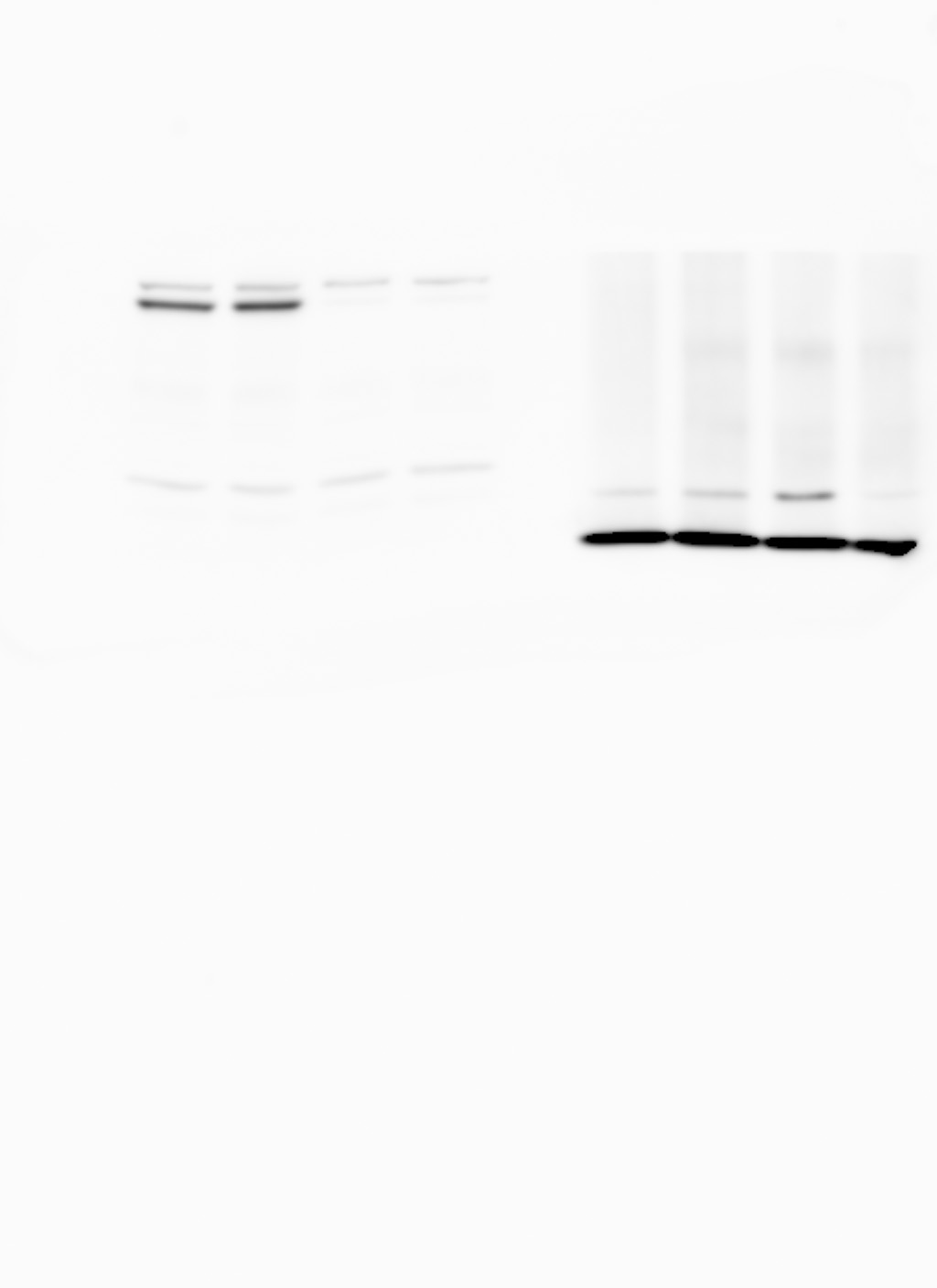

Supplement: Source data 1. [file elife-69705-data1.zip › JPEG/Figure1H-SourceData1-TELO2.jpg]

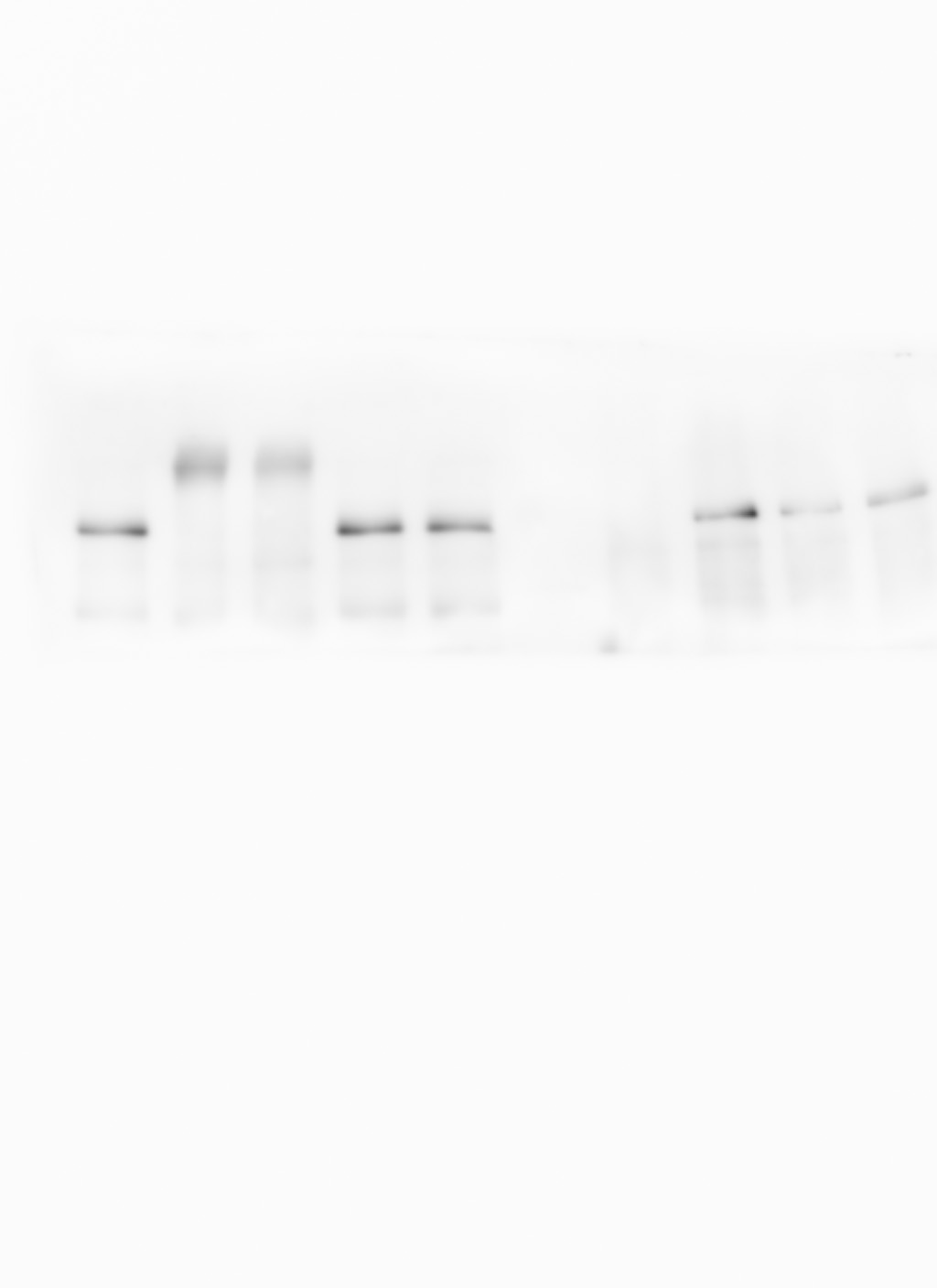

Supplement: Source data 1. [file elife-69705-data1.zip › JPEG/Figure1H-SourceData1-TRRAP.jpg]

H

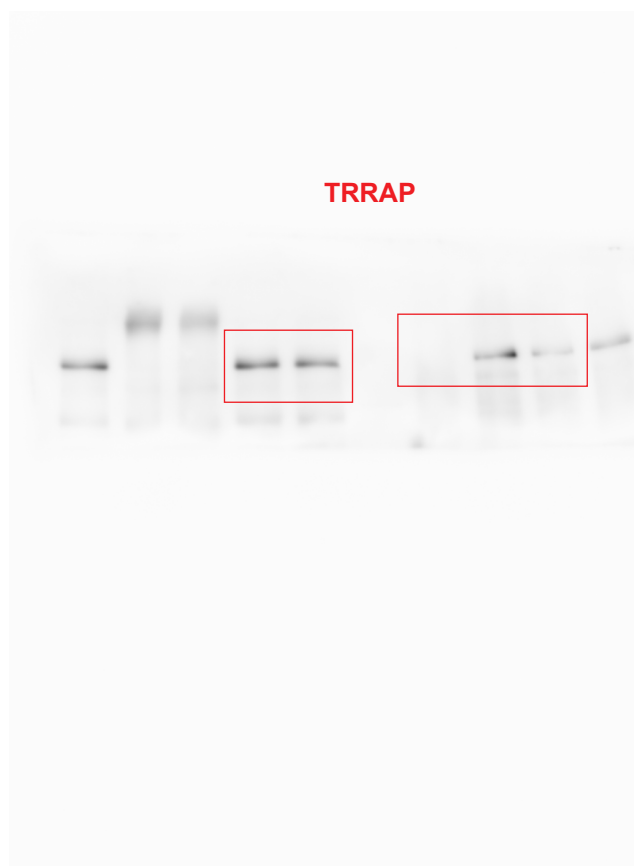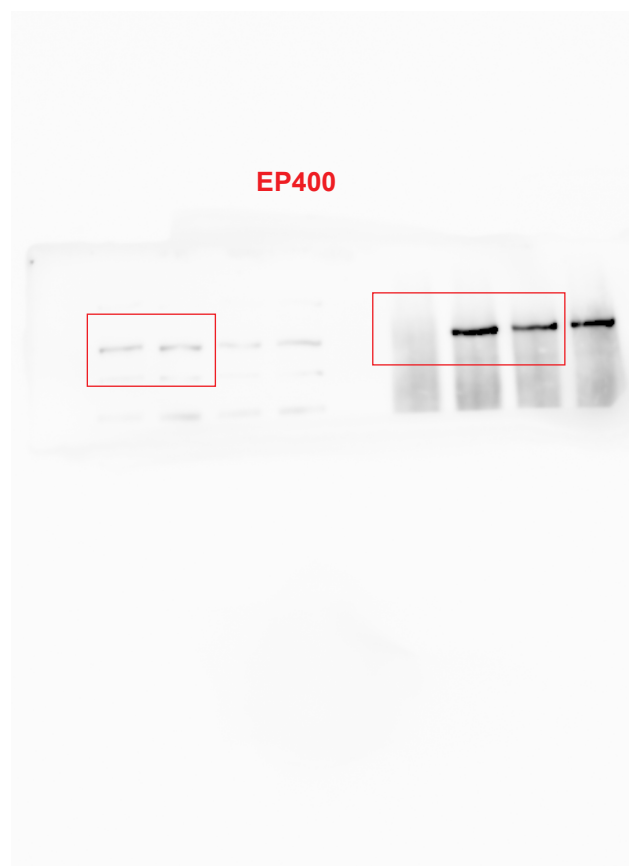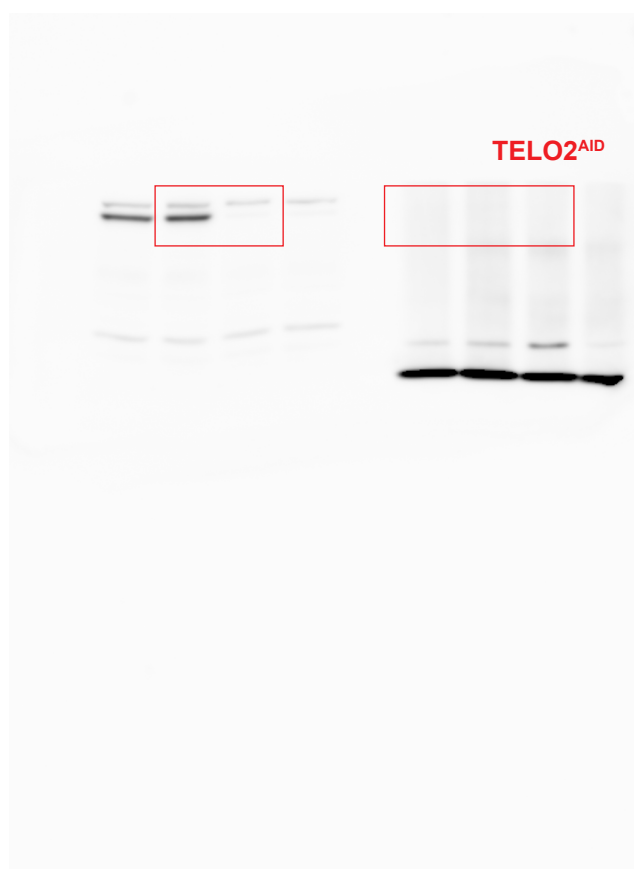

Supplement: Source data 1. [file elife-69705-data1.zip › JPEG/Figure1H-SourceData1.pdf]

**B**

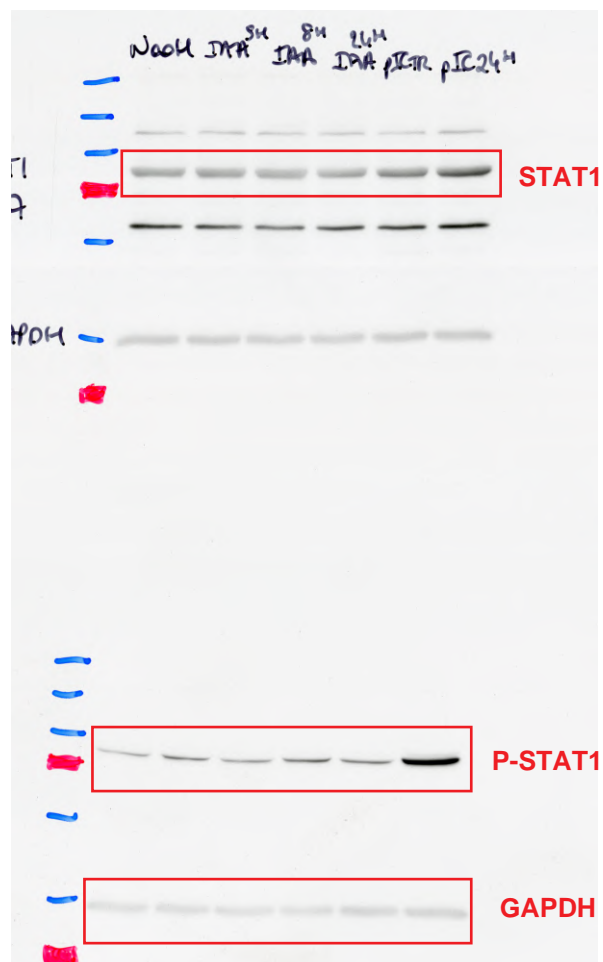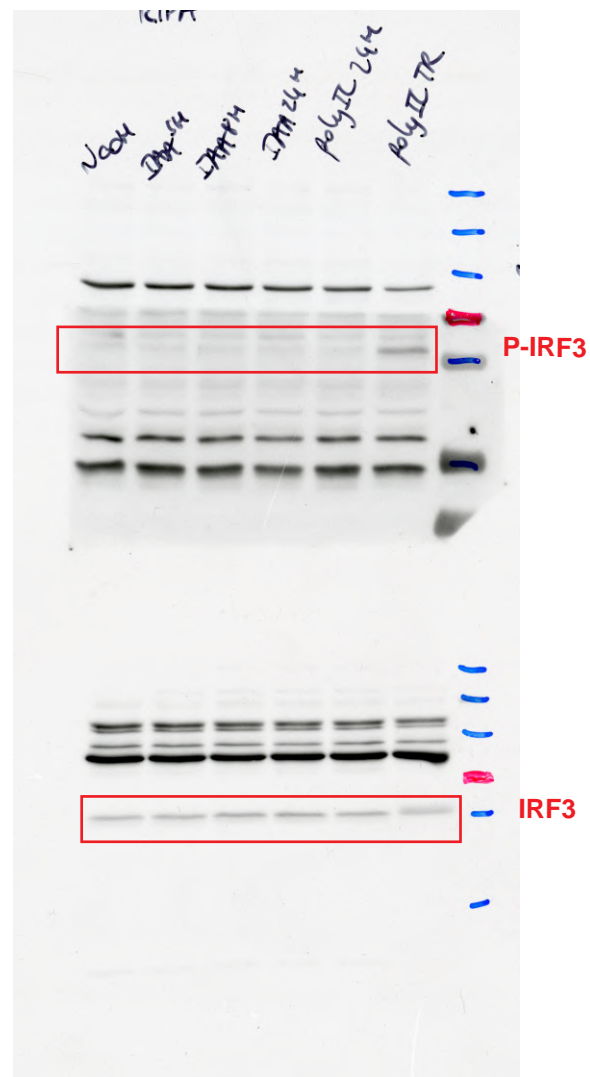

Supplement: Source data 1. [file elife-69705-data1.zip › JPEG/Figure3-SourceData1B_compressed.pdf]

C

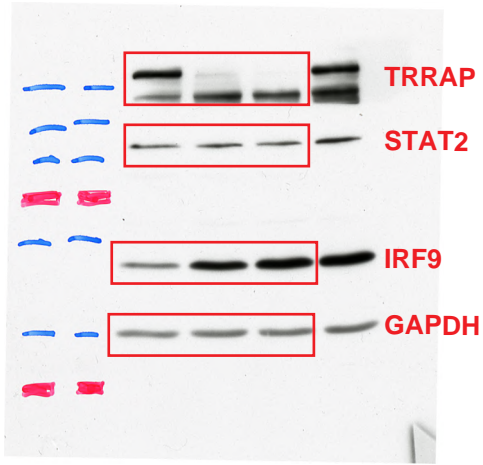

D

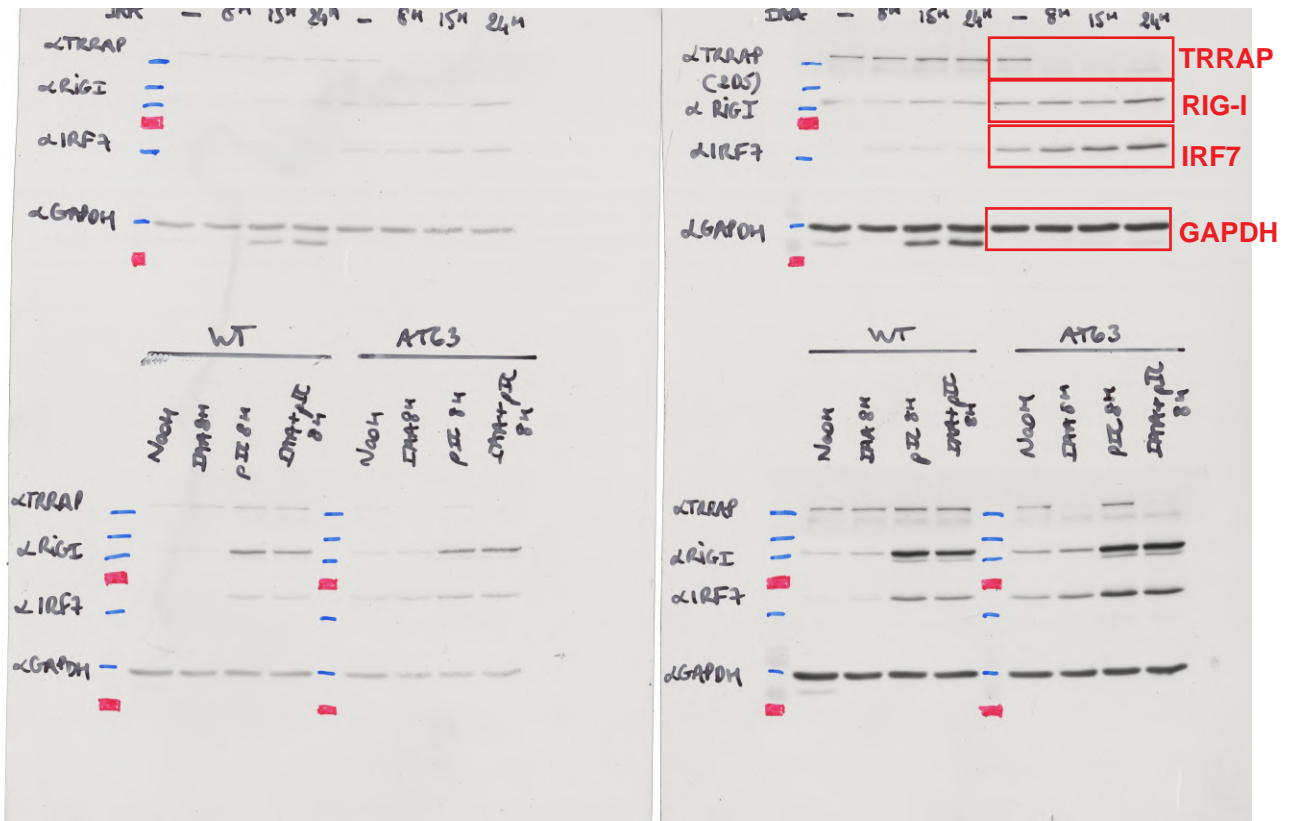

Supplement: Source data 1. [file elife-69705-data1.zip › JPEG/Figure3-SourceData1C-D_compressed.pdf]

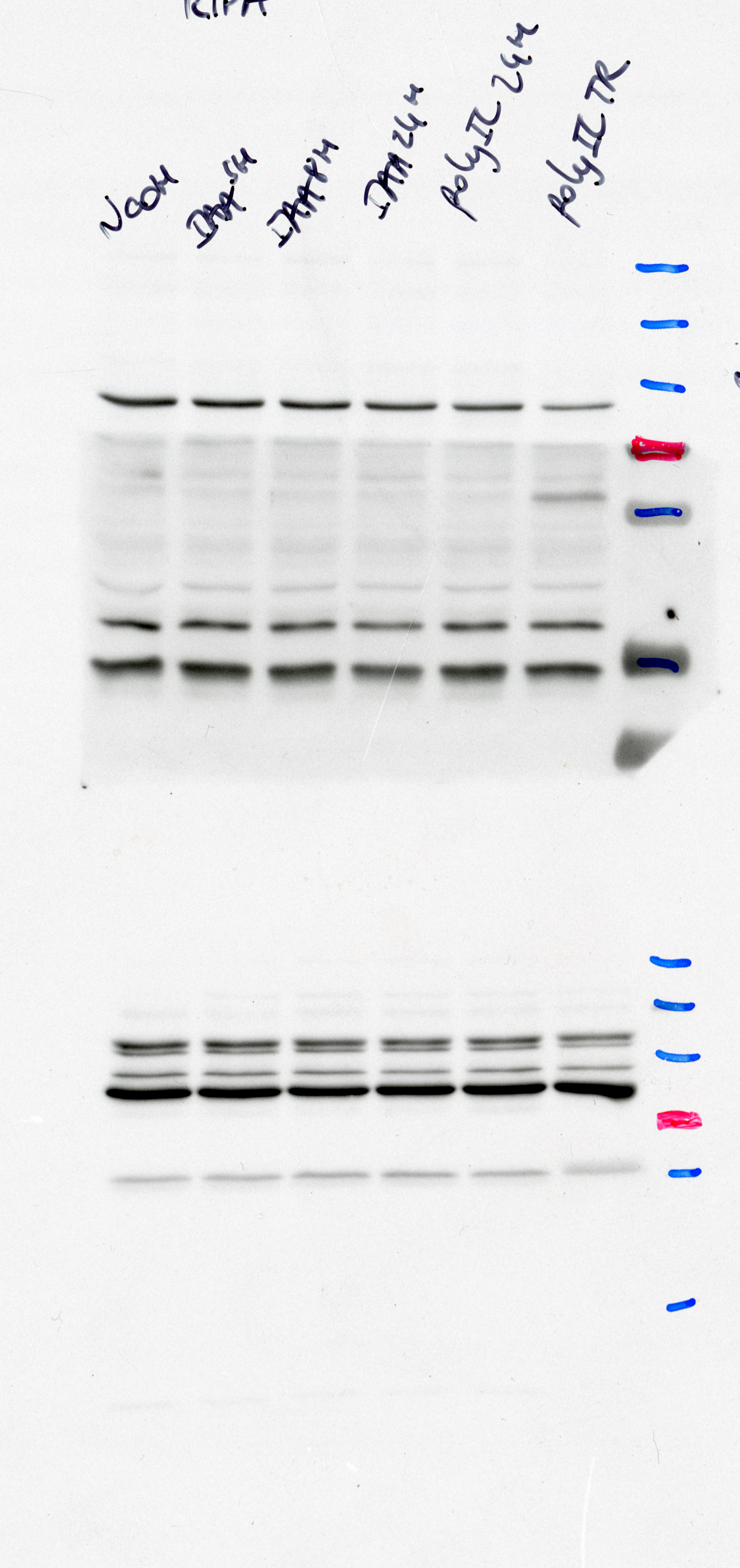

Supplement: Source data 1. [file elife-69705-data1.zip › JPEG/Figure3B-SourceData1-IRF3.jpg]

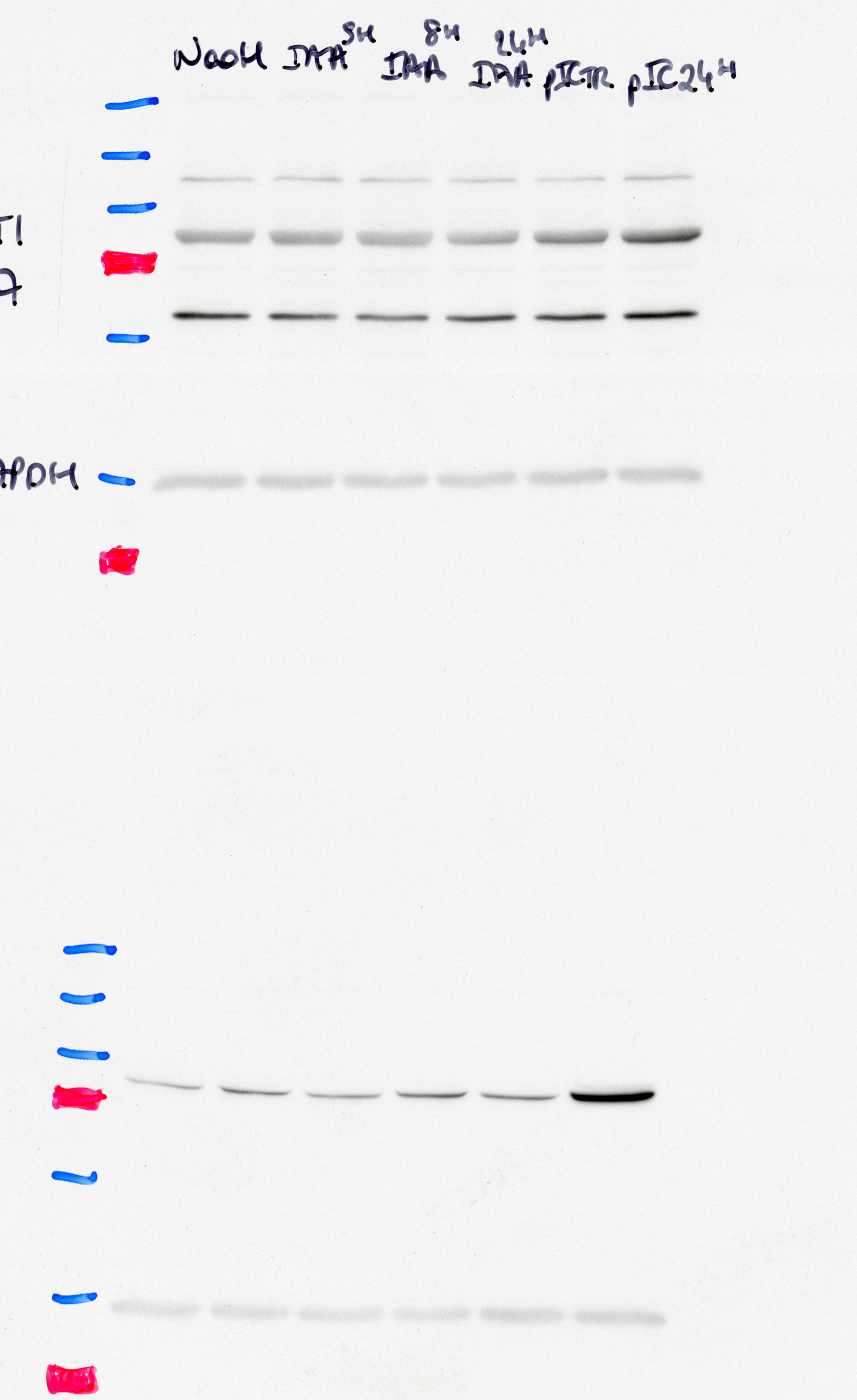

Supplement: Source data 1. [file elife-69705-data1.zip › JPEG/Figure3B-SourceData1-STAT1.jpg]

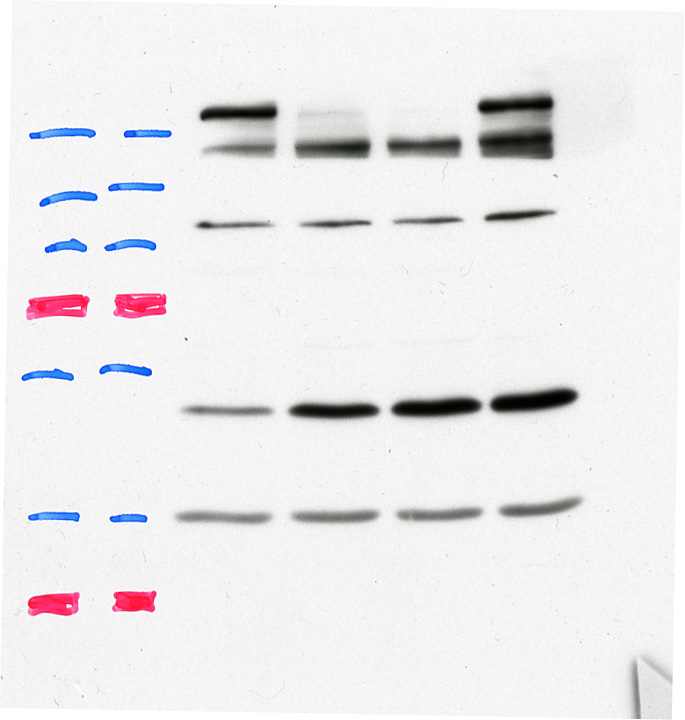

Supplement: Source data 1. [file elife-69705-data1.zip › JPEG/Figure3C-SourceData1.jpg]

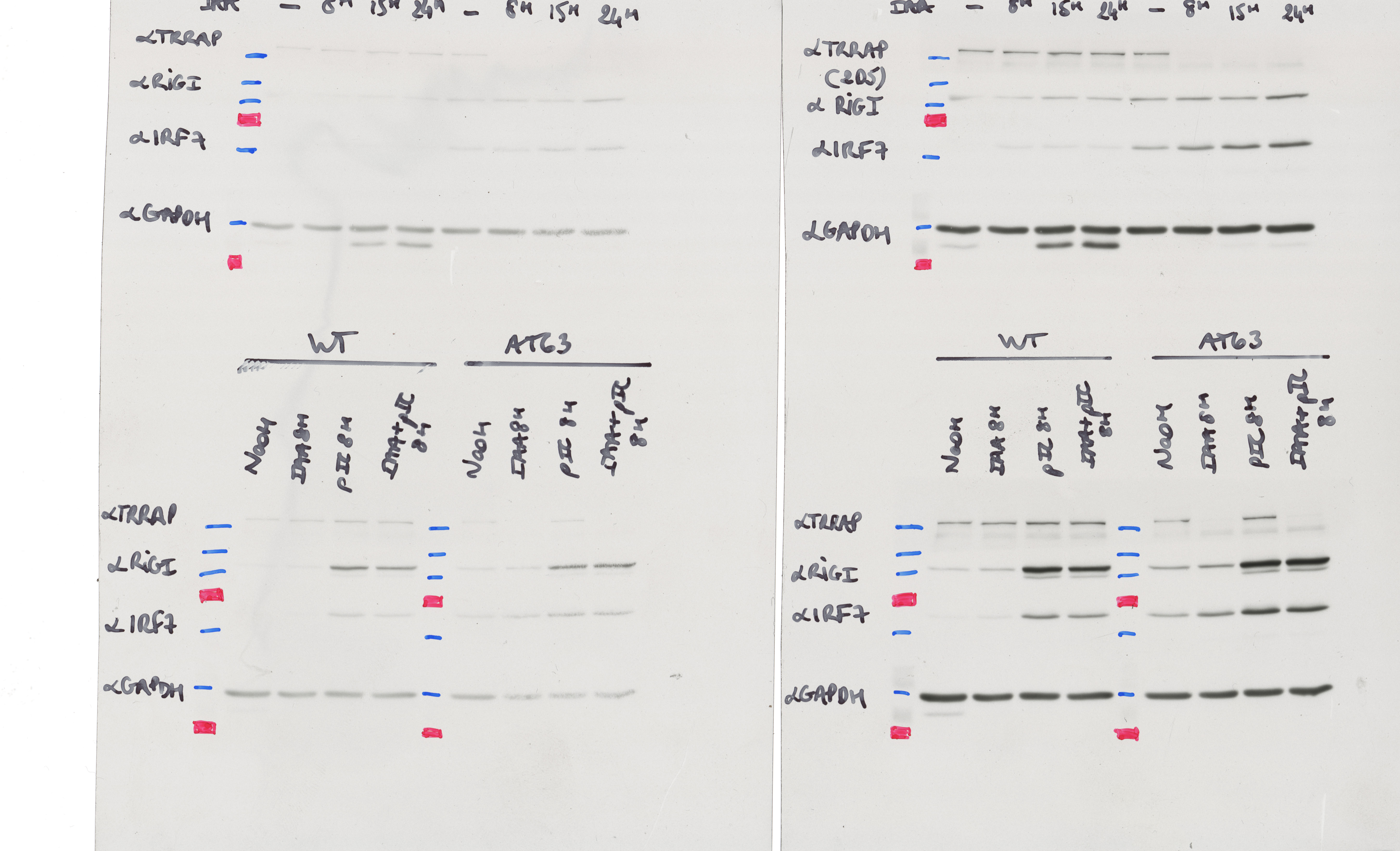

Supplement: Source data 1. [file elife-69705-data1.zip › JPEG/Figure3D-SourceData1.jpg]

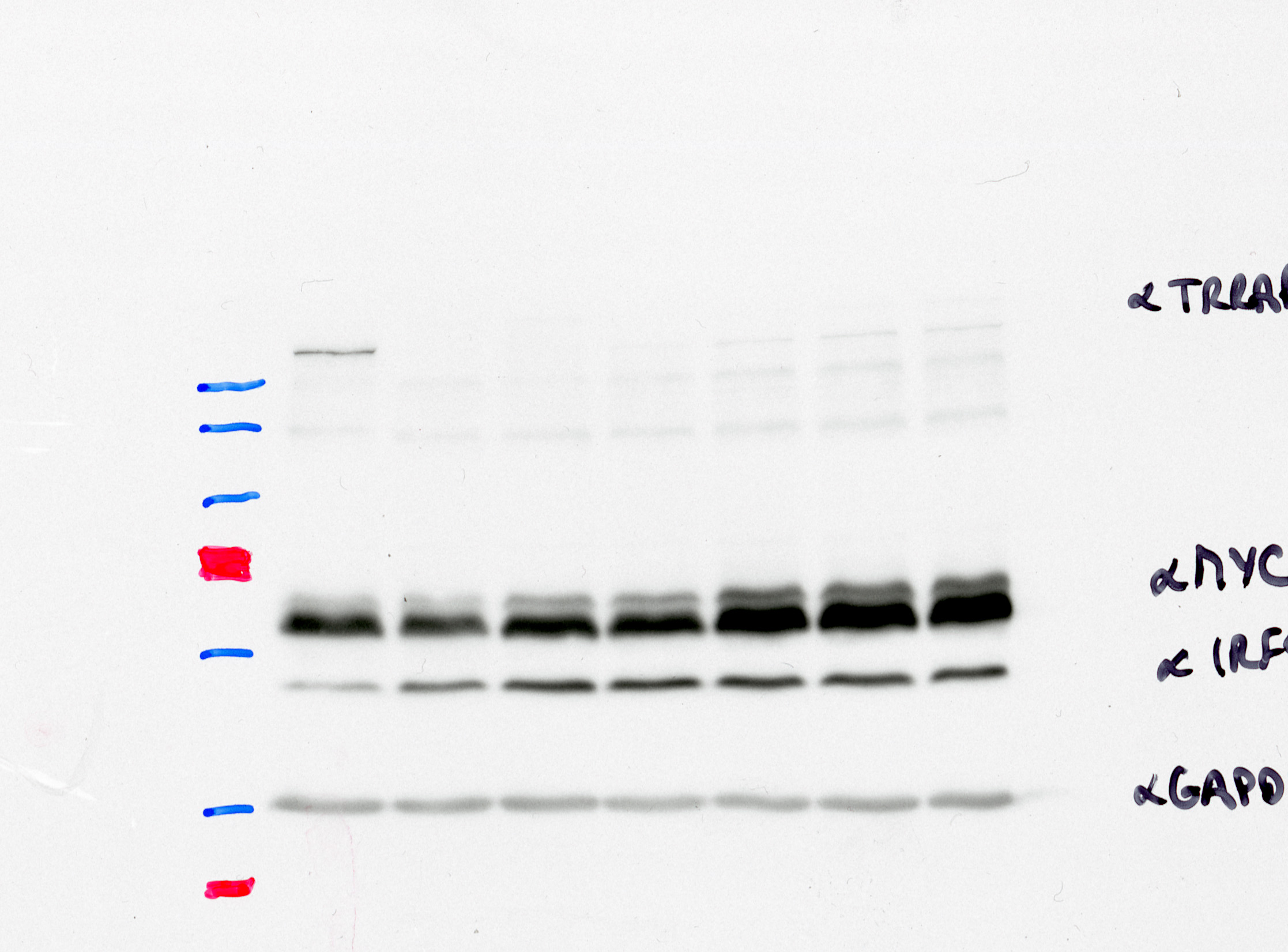

Supplement: Source data 1. [file elife-69705-data1.zip › JPEG/Figure7B-SourceData1short.jpg]

**B**

Long exposure

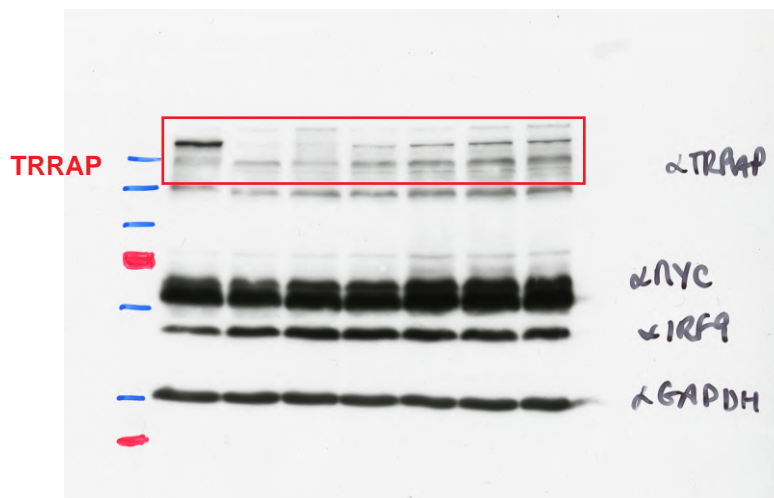

Short exposure

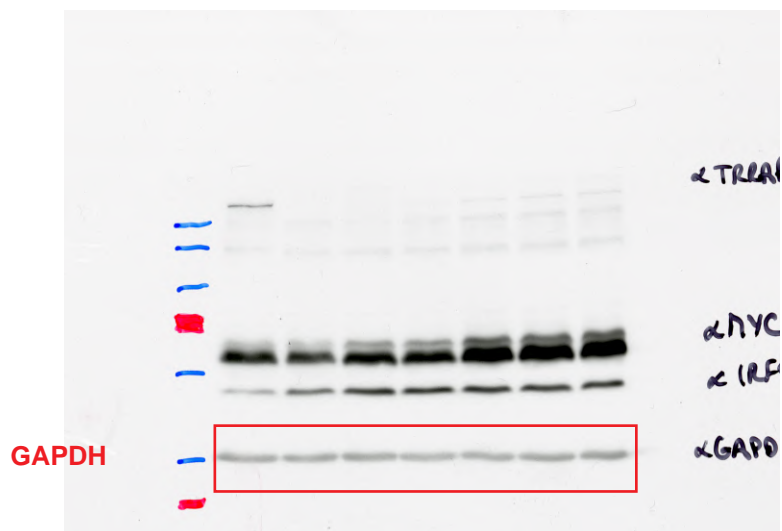

Supplement: Source data 1. [file elife-69705-data1.zip › JPEG/Figure7B-SourceData1_compressed.pdf]

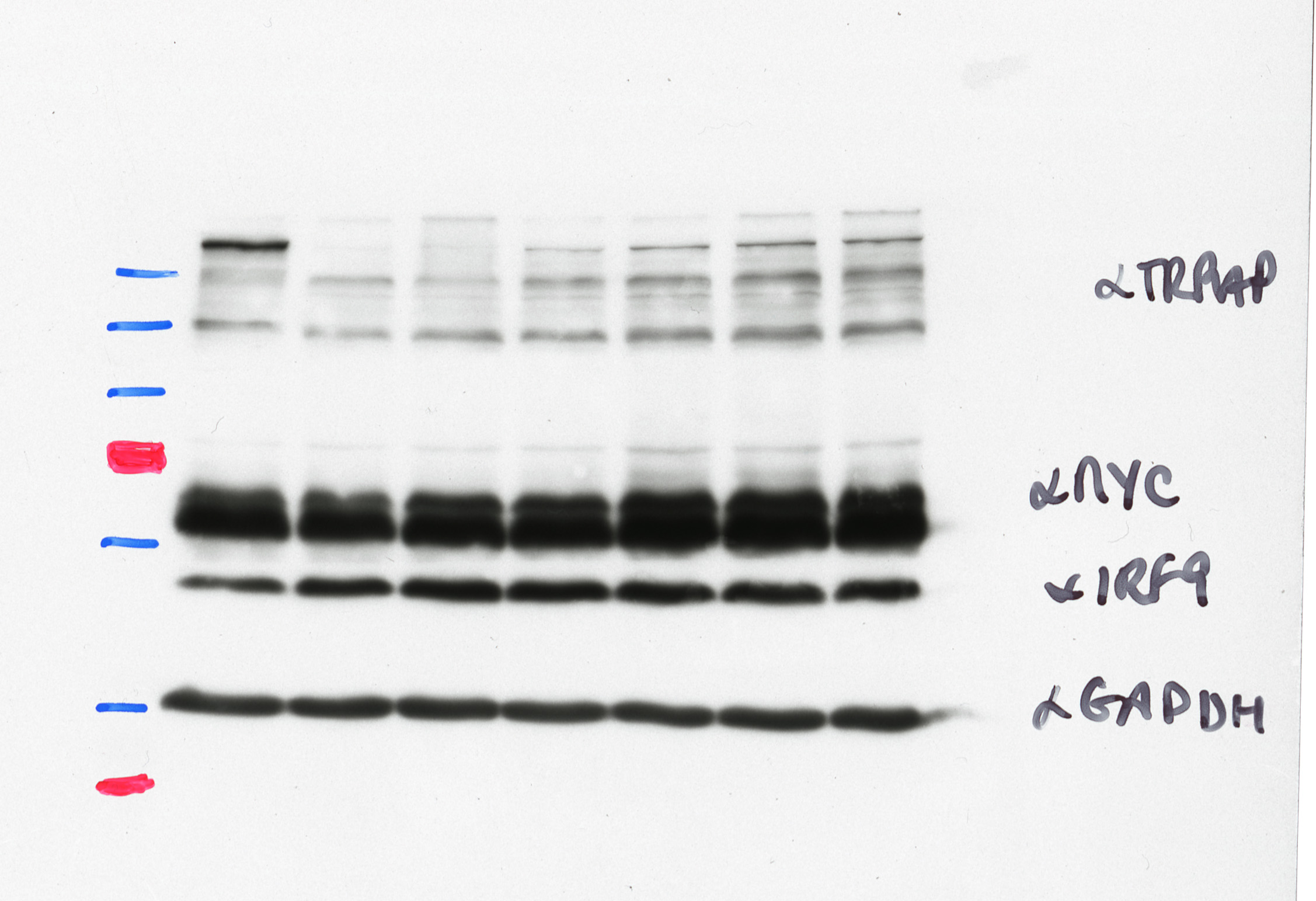

Supplement: Source data 1. [file elife-69705-data1.zip › JPEG/Figure7B-SourceData2long.jpg]

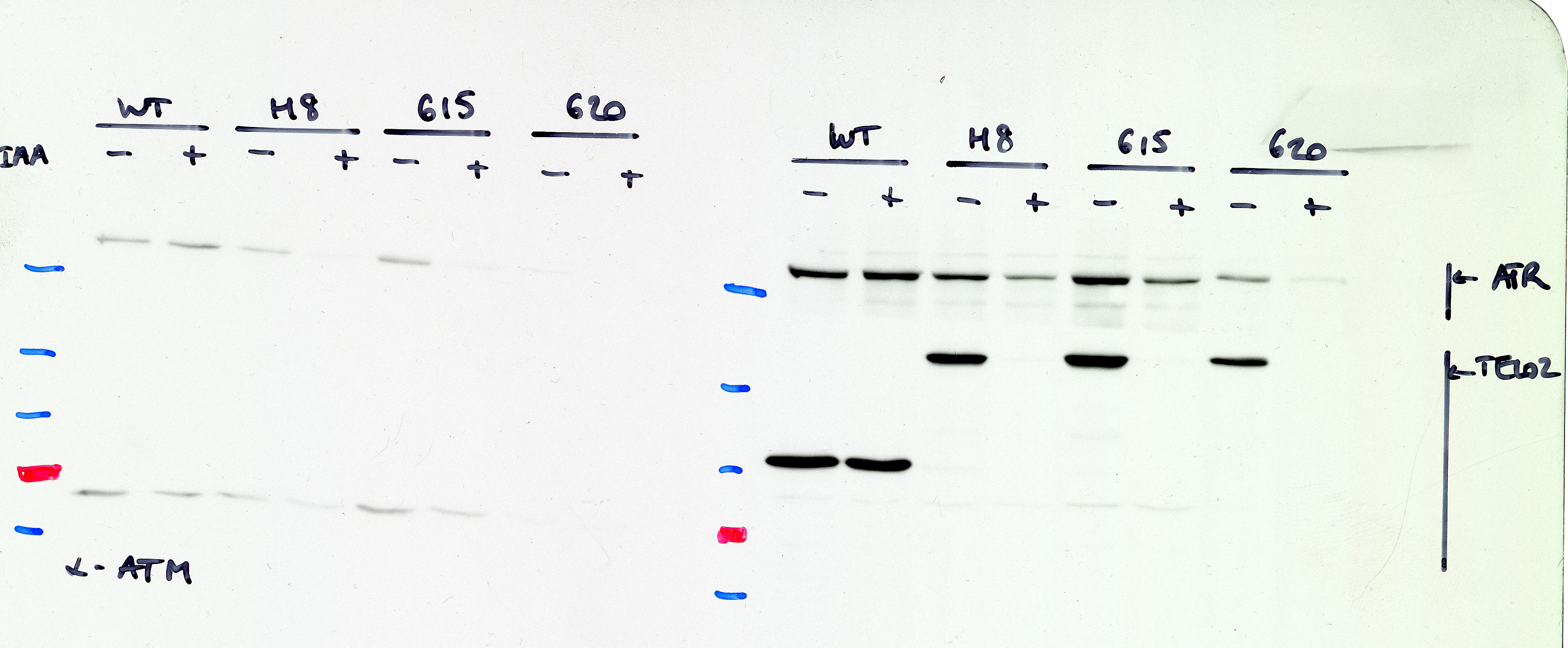

Supplement: Source data 1. [file elife-69705-data1.zip › JPEG/Figure 1-figure supplement 1-SourceData1-ATM-ATR.jpg]

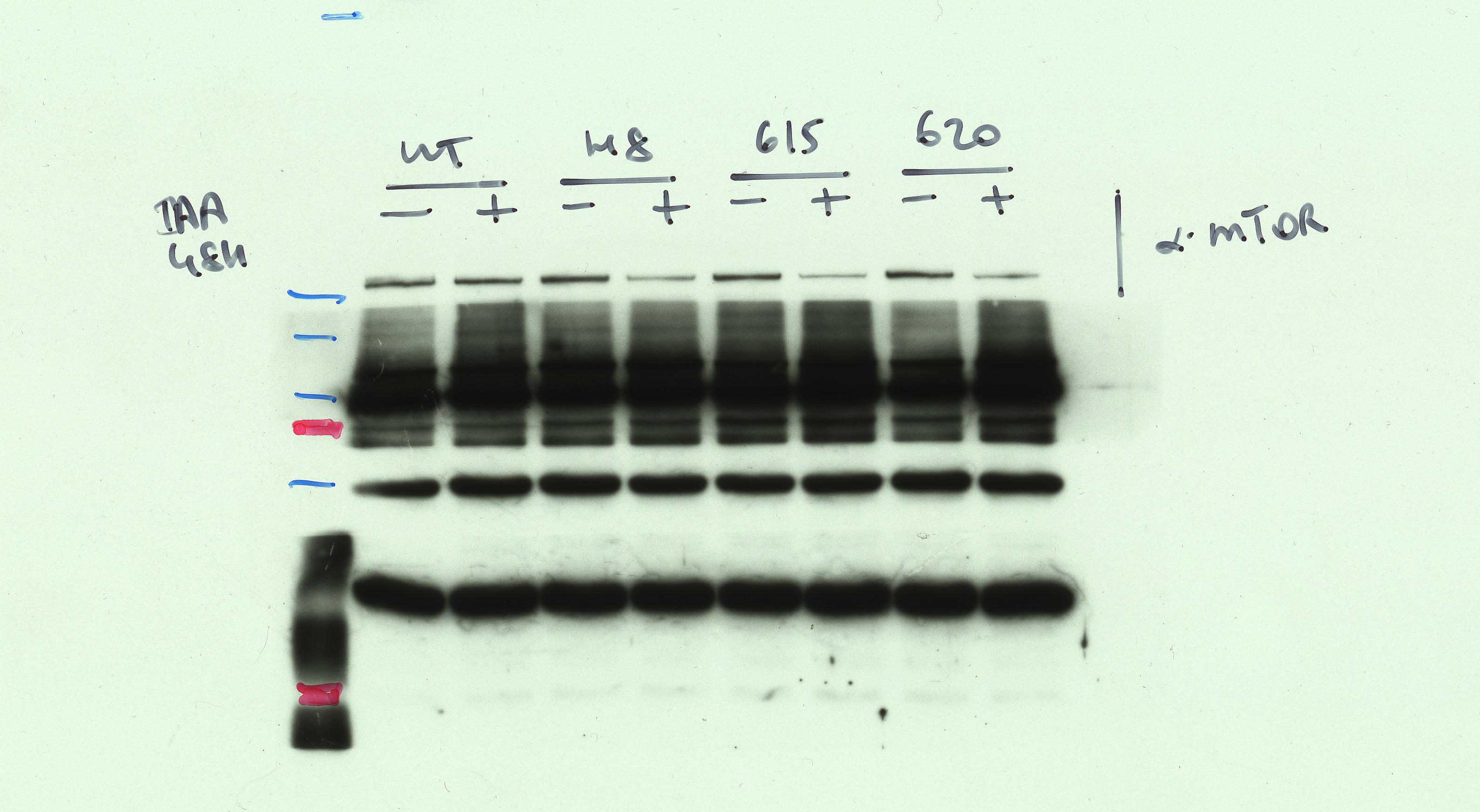

Supplement: Source data 1. [file elife-69705-data1.zip › JPEG/Figure 1-figure supplement 1-SourceData1-MTOR.jpg]

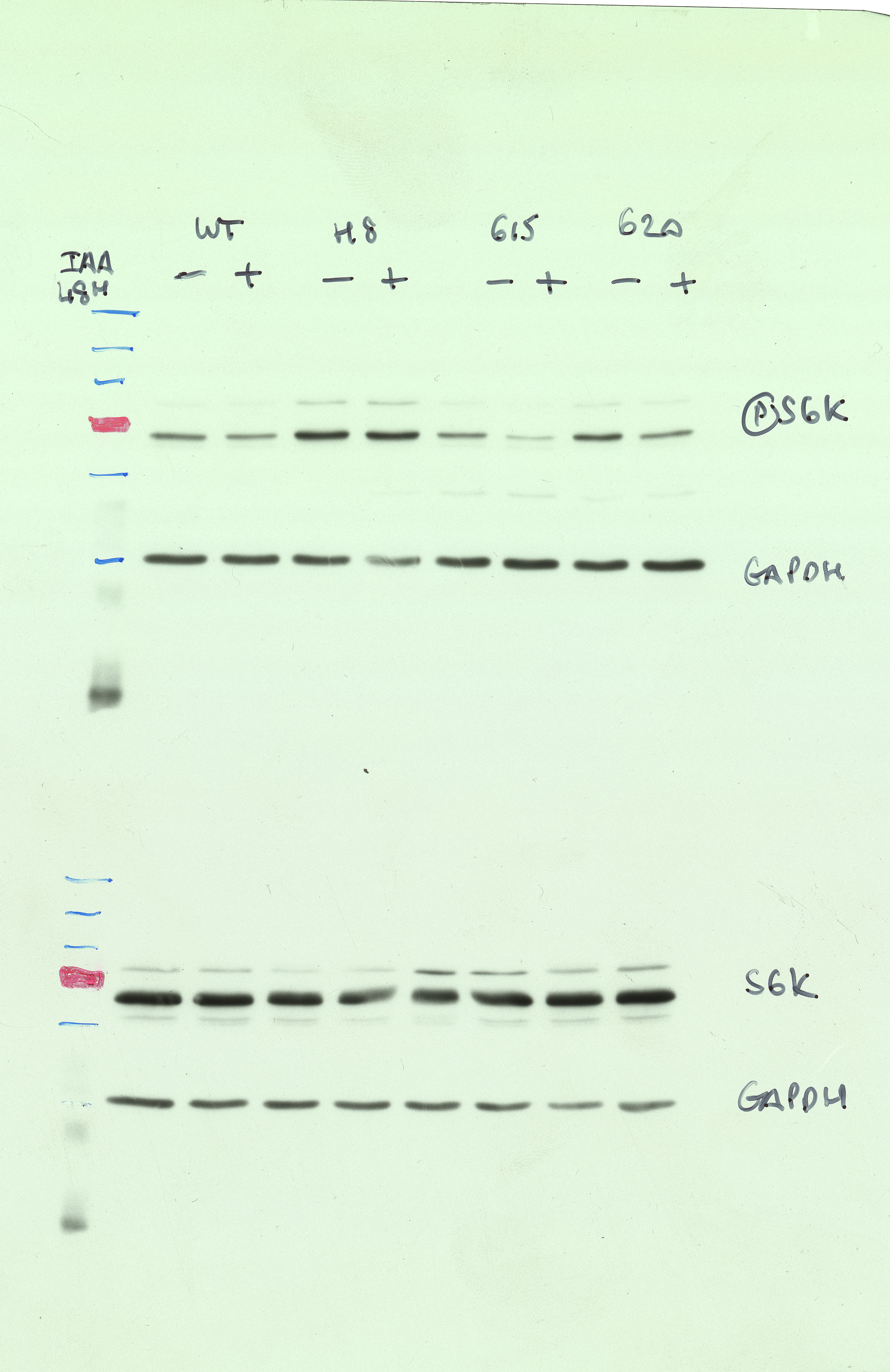

Supplement: Source data 1. [file elife-69705-data1.zip › JPEG/Figure 1-figure supplement 1-SourceData1-S6K-pS6K-GAPDH.jpg]

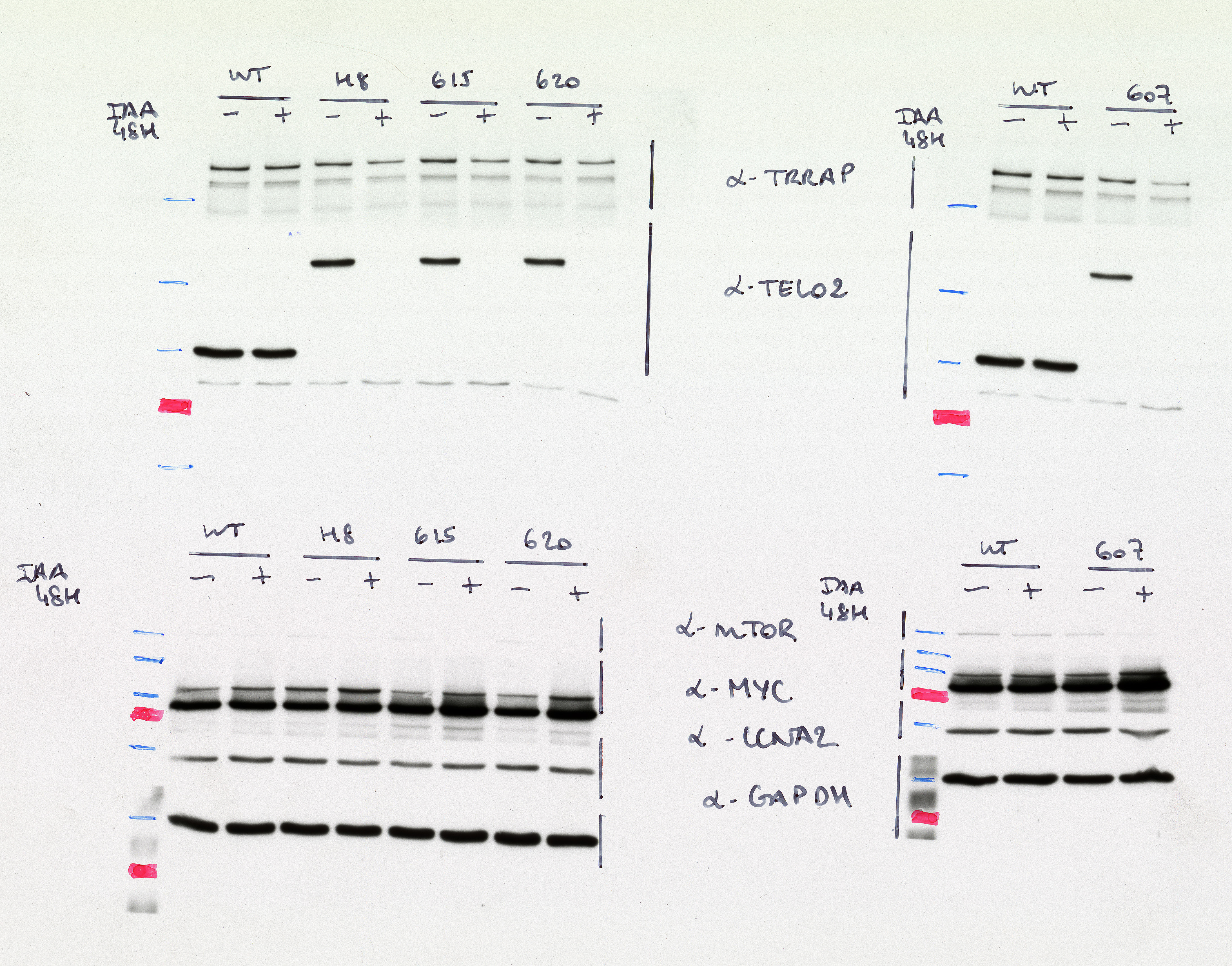

Supplement: Source data 1. [file elife-69705-data1.zip › JPEG/Figure 1-figure supplement 1-SourceData1-TRRAP-TELO2-TIR1-GAPDH-long.jpg]

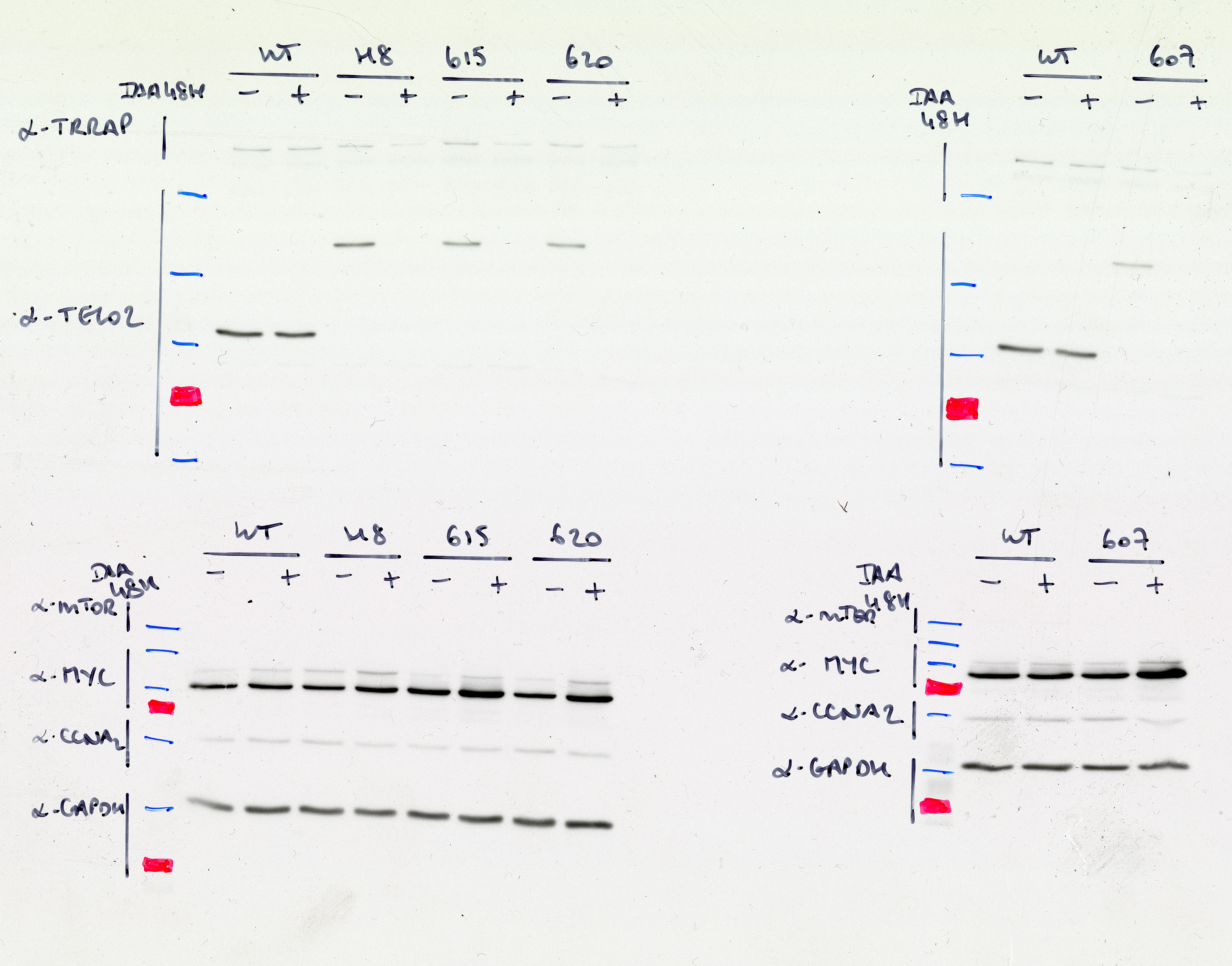

Supplement: Source data 1. [file elife-69705-data1.zip › JPEG/Figure 1-figure supplement 1-SourceData1-TRRAP-TELO2-TIR1-GAPDH-short.jpg]

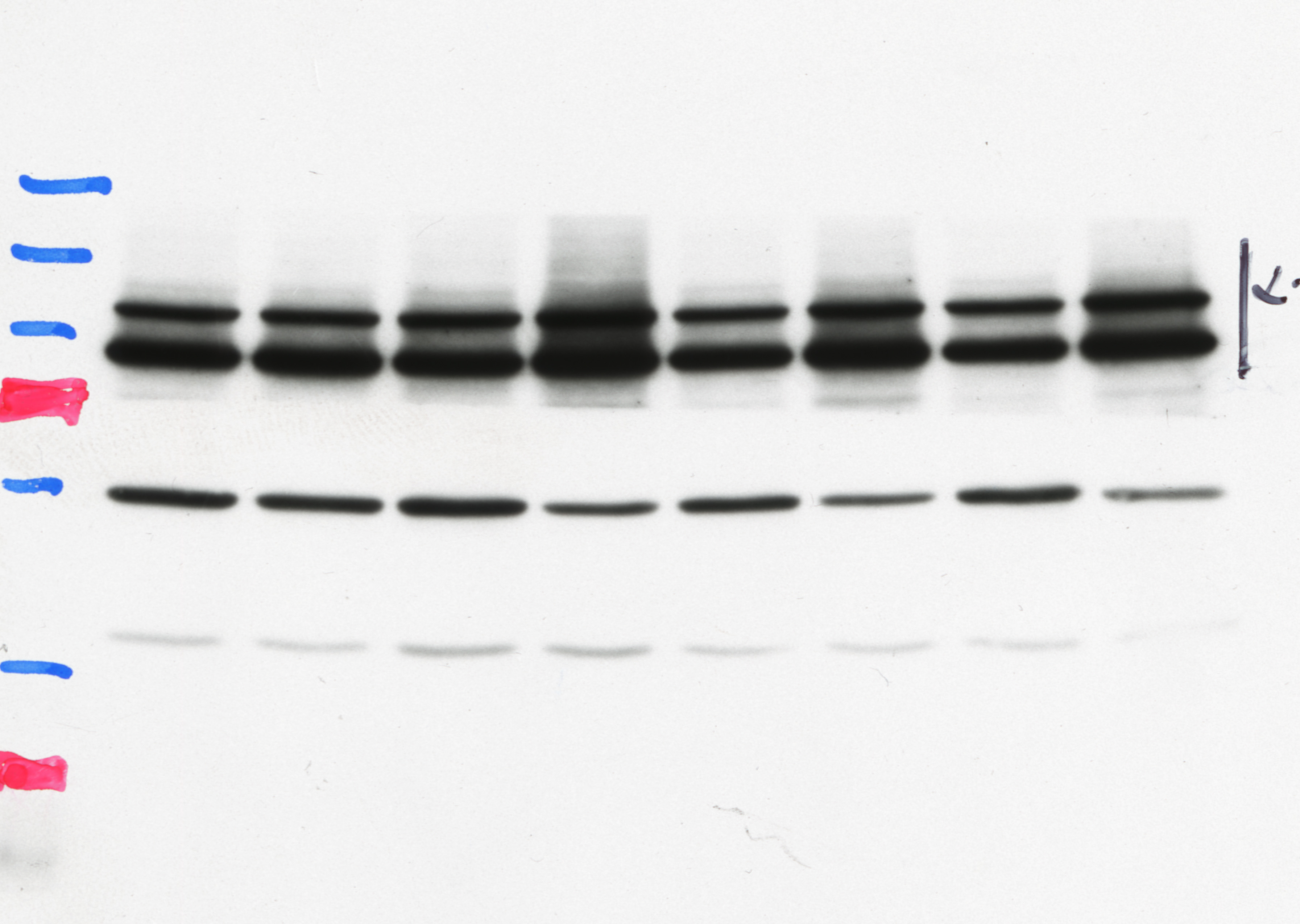

Supplement: Source data 1. [file elife-69705-data1.zip › JPEG/Figure 1-figure supplement 1-SourceData1-CCNA2.jpg]

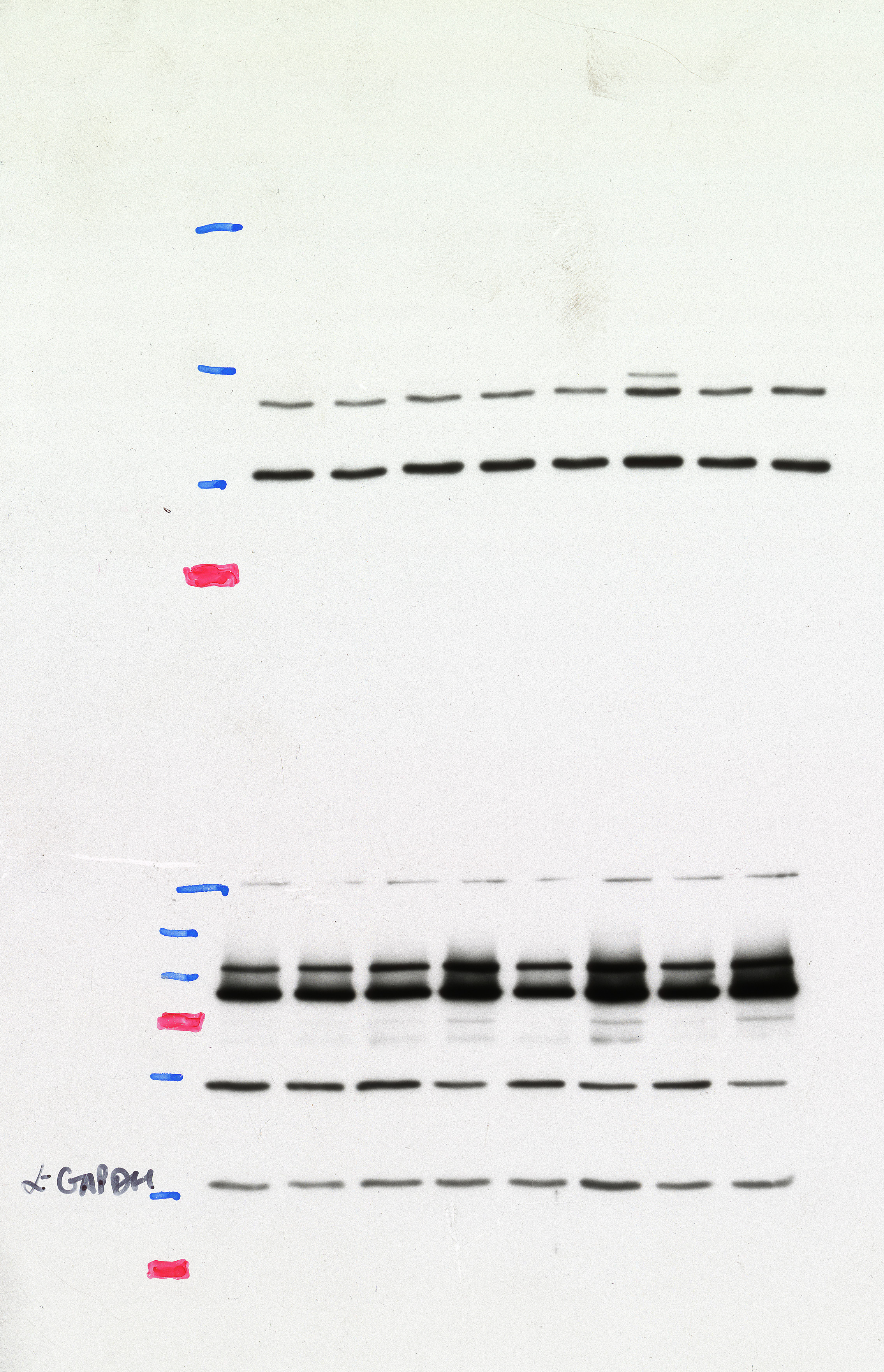

Supplement: Source data 1. [file elife-69705-data1.zip › JPEG/Figure 1-figure supplement 1-SourceData1-GAPDH.jpg]

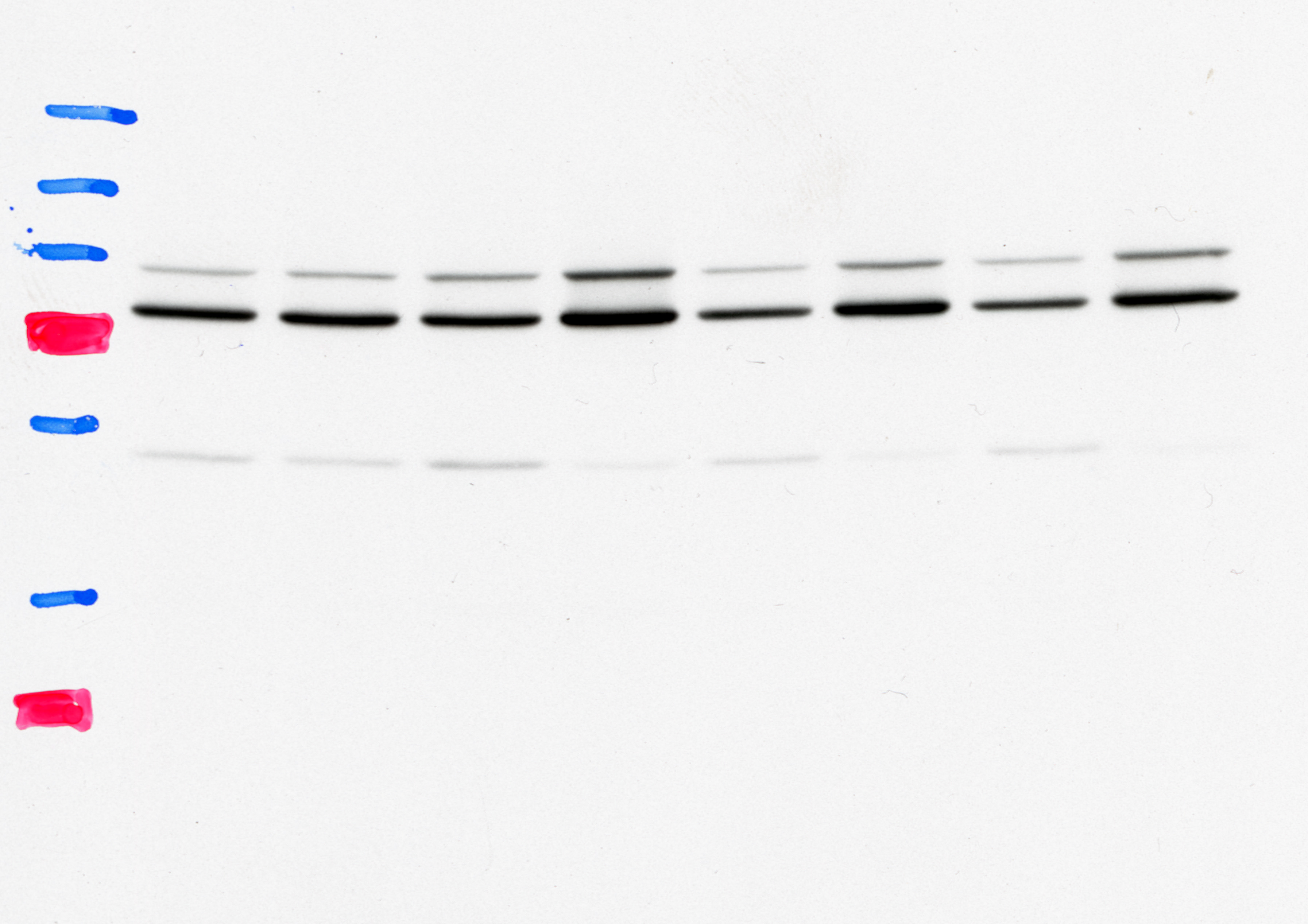

Supplement: Source data 1. [file elife-69705-data1.zip › JPEG/Figure 1-figure supplement 1-SourceData1-TIR.jpg]

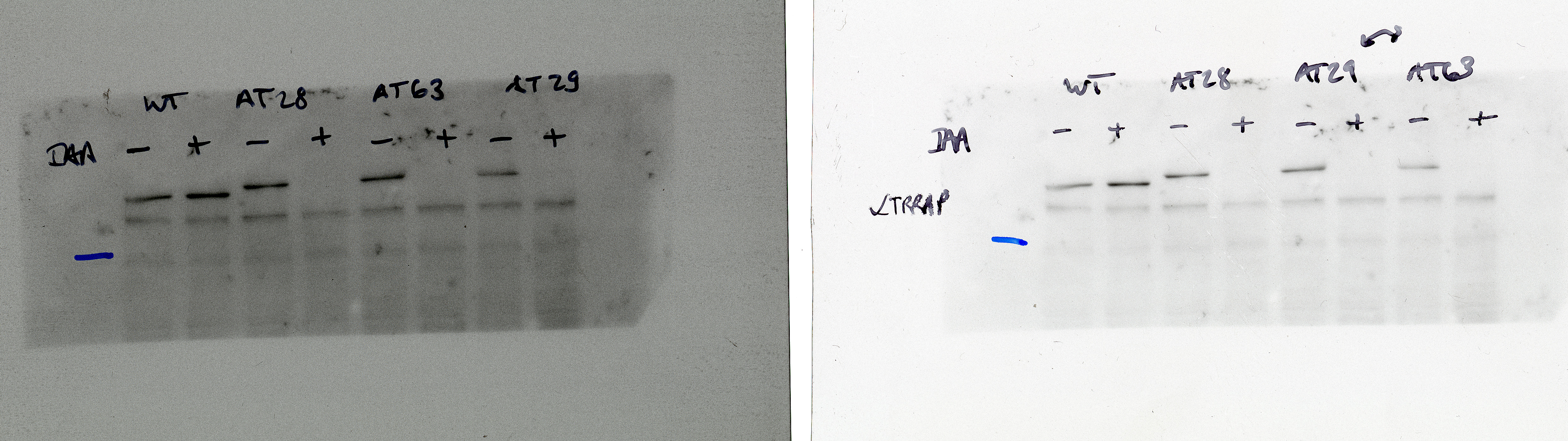

Supplement: Source data 1. [file elife-69705-data1.zip › JPEG/Figure 1-figure supplement 1-SourceData1-TRRAP.jpg]

E

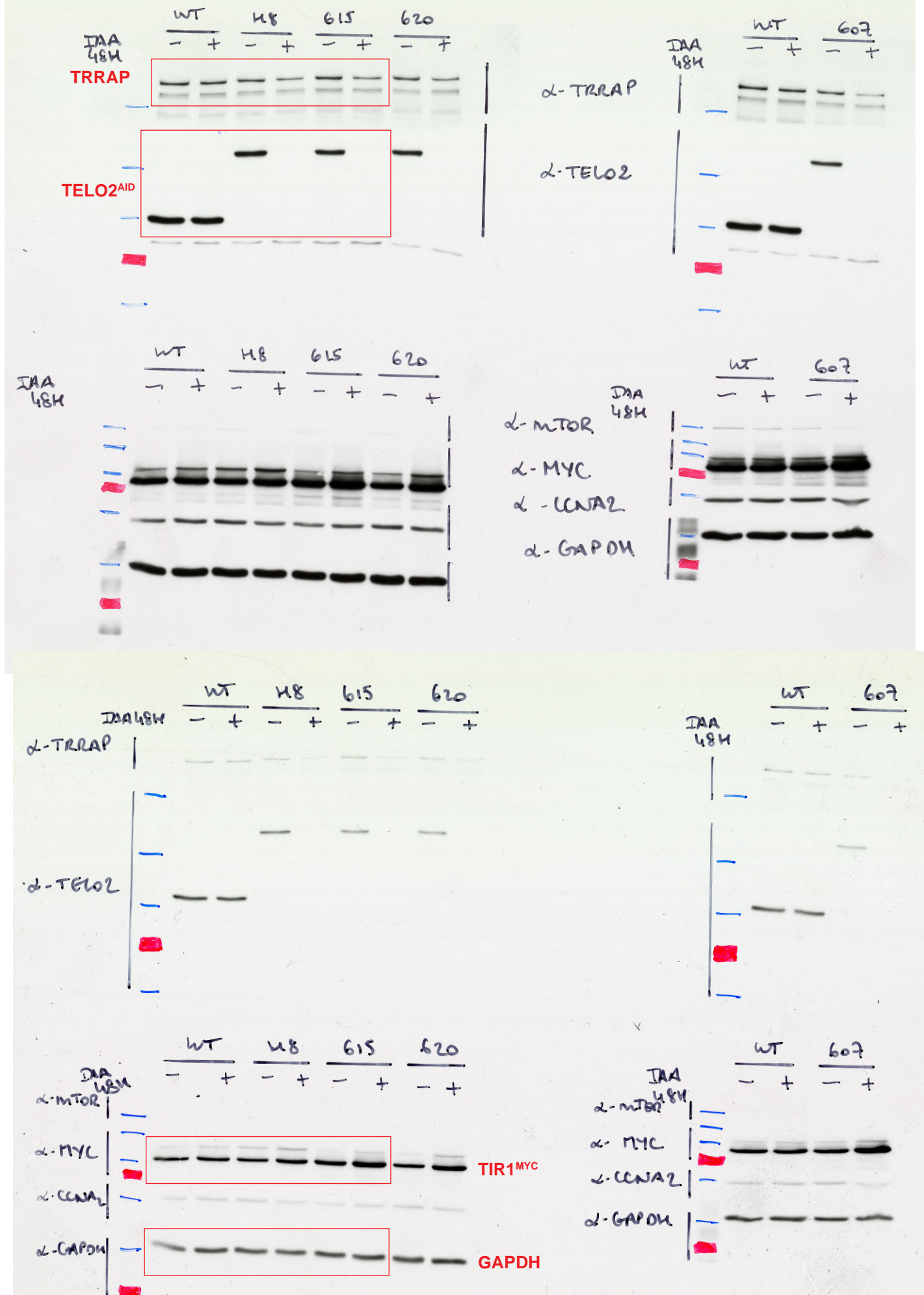

Supplement: Source data 1. [file elife-69705-data1.zip › JPEG/Figure 1-figure supplement 1-SourceData1_compressed.pdf]

E

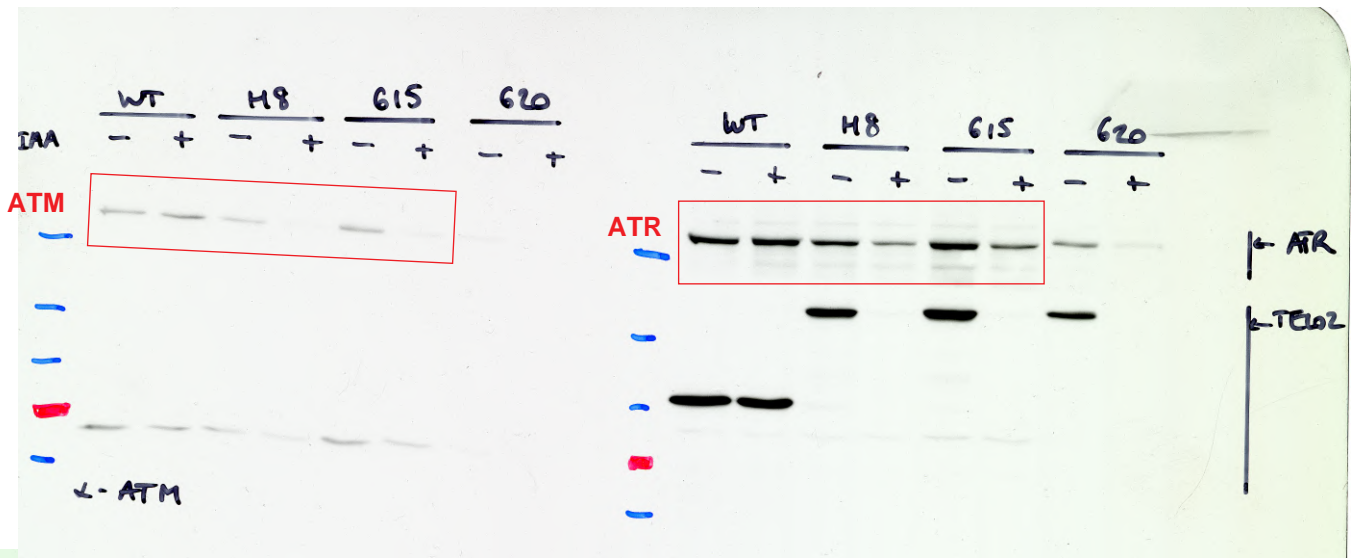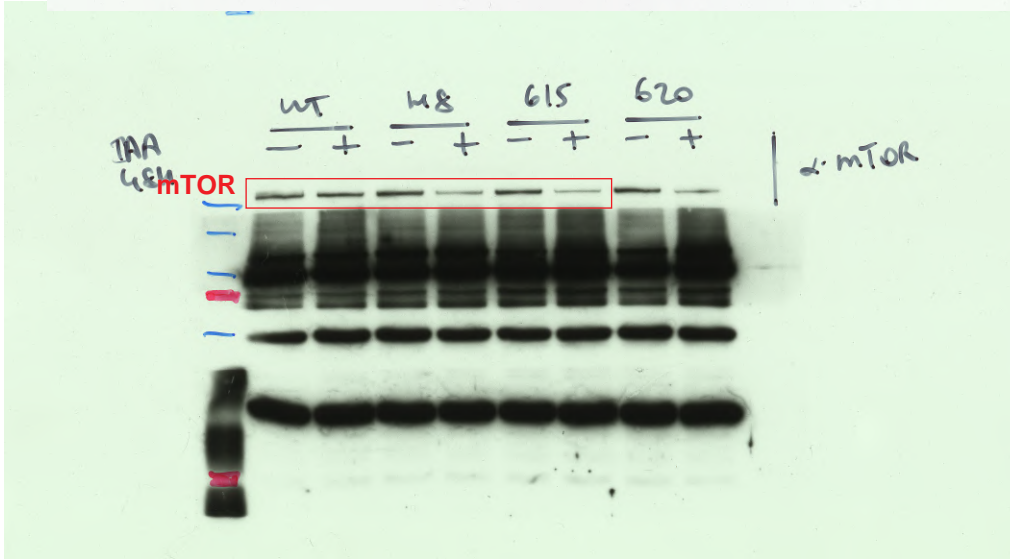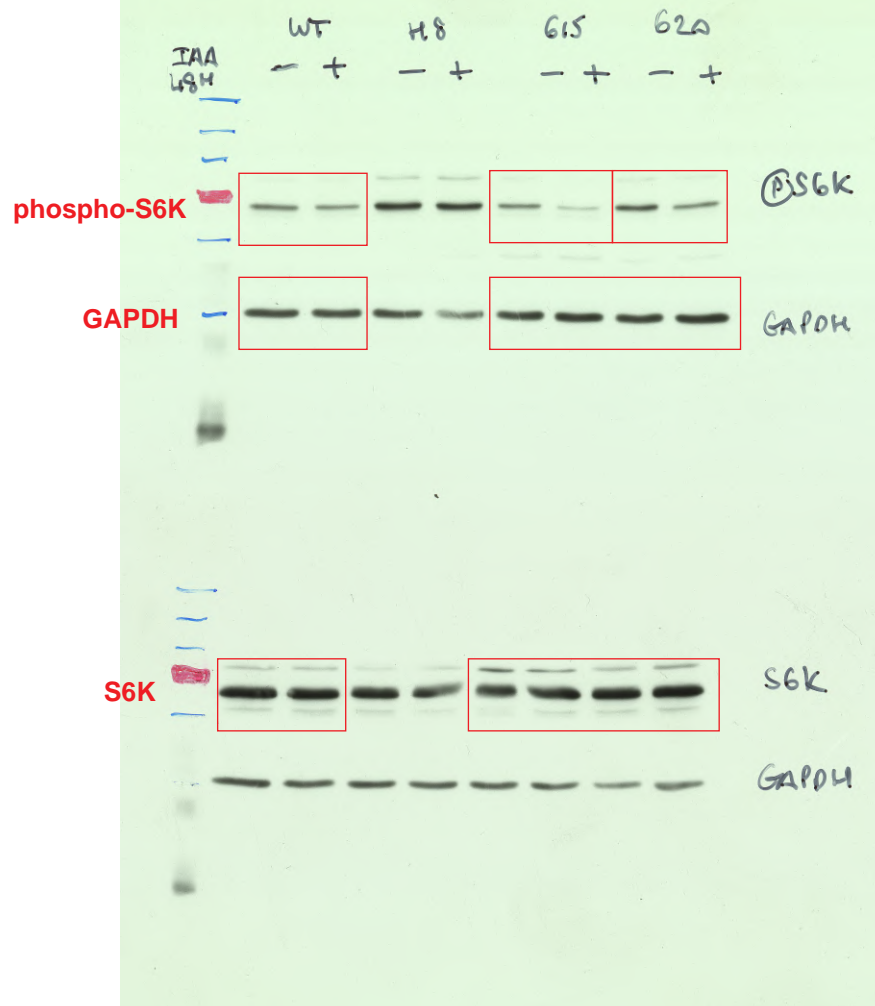

Supplement: Source data 1. [file elife-69705-data1.zip › JPEG/Figure 1-figure supplement 1-SourceData2_compressed.pdf]

F

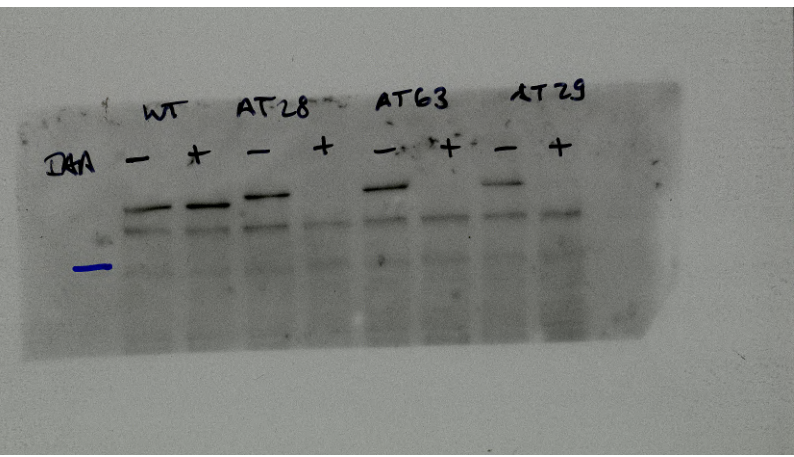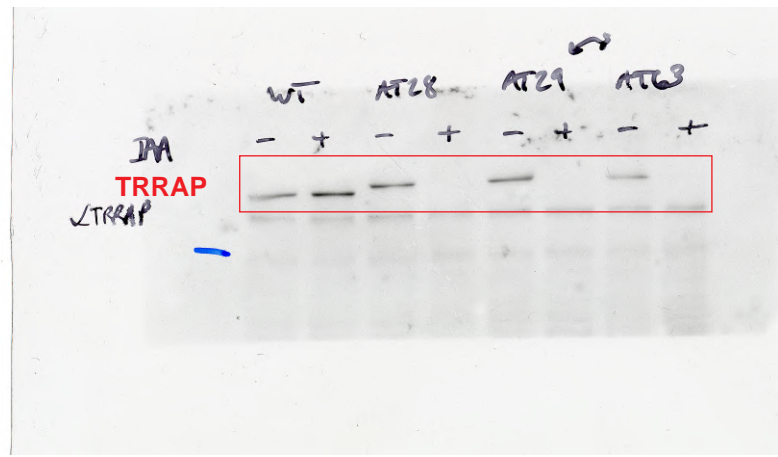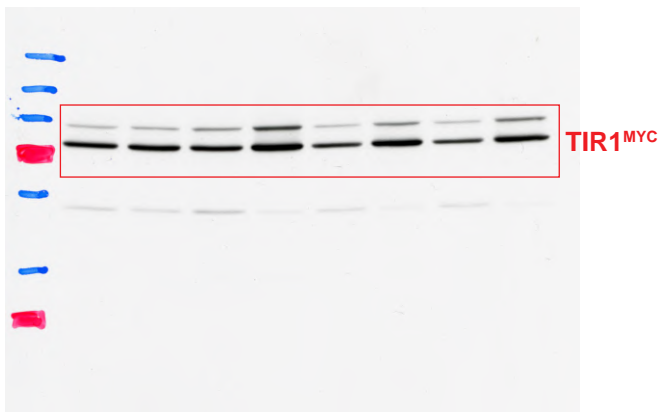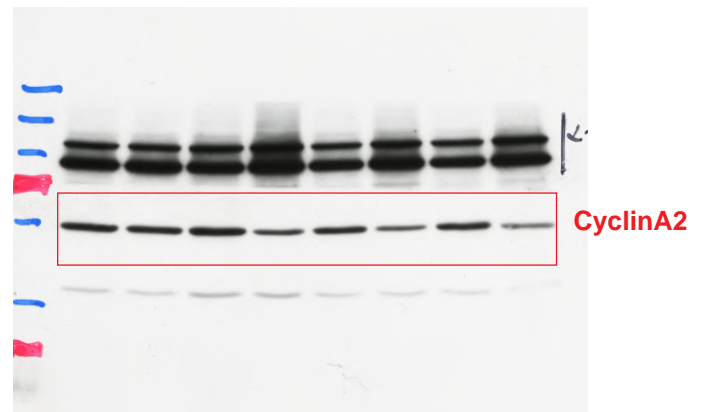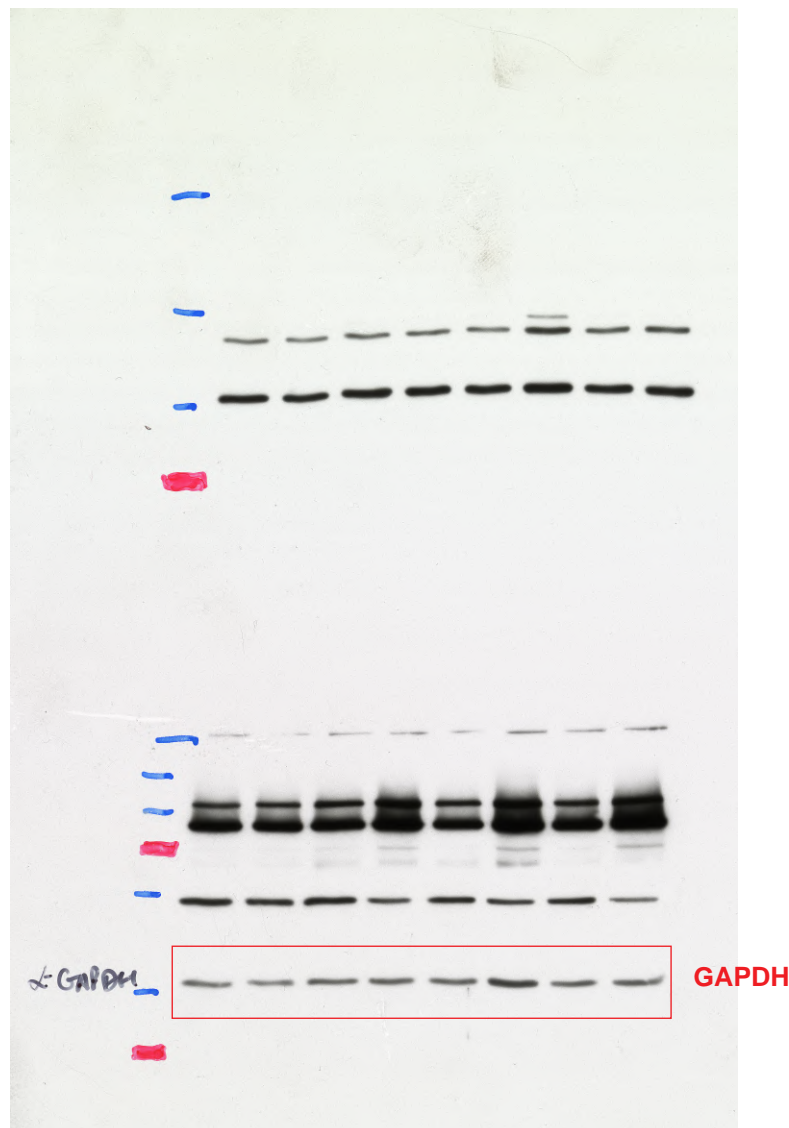

Supplement: Source data 1. [file elife-69705-data1.zip › JPEG/Figure 1-figure supplement 1-SourceData1_compressed.pdf]
