# Supplementary material for: The TRRAP transcription cofactor represses interferon-stimulated genes in colorectal cancer cells
Source: eLife. 2022 Mar 4;11:e69705. doi: 10.7554/eLife.69705 (PMC8926402; doi:10.7554/eLife.69705)
Supplement: Source data 1. [file Figure1E-F-SourceData1_compressed.pdf › JPEG/Figure1E-F-SourceData1_compressed.pdf]

E

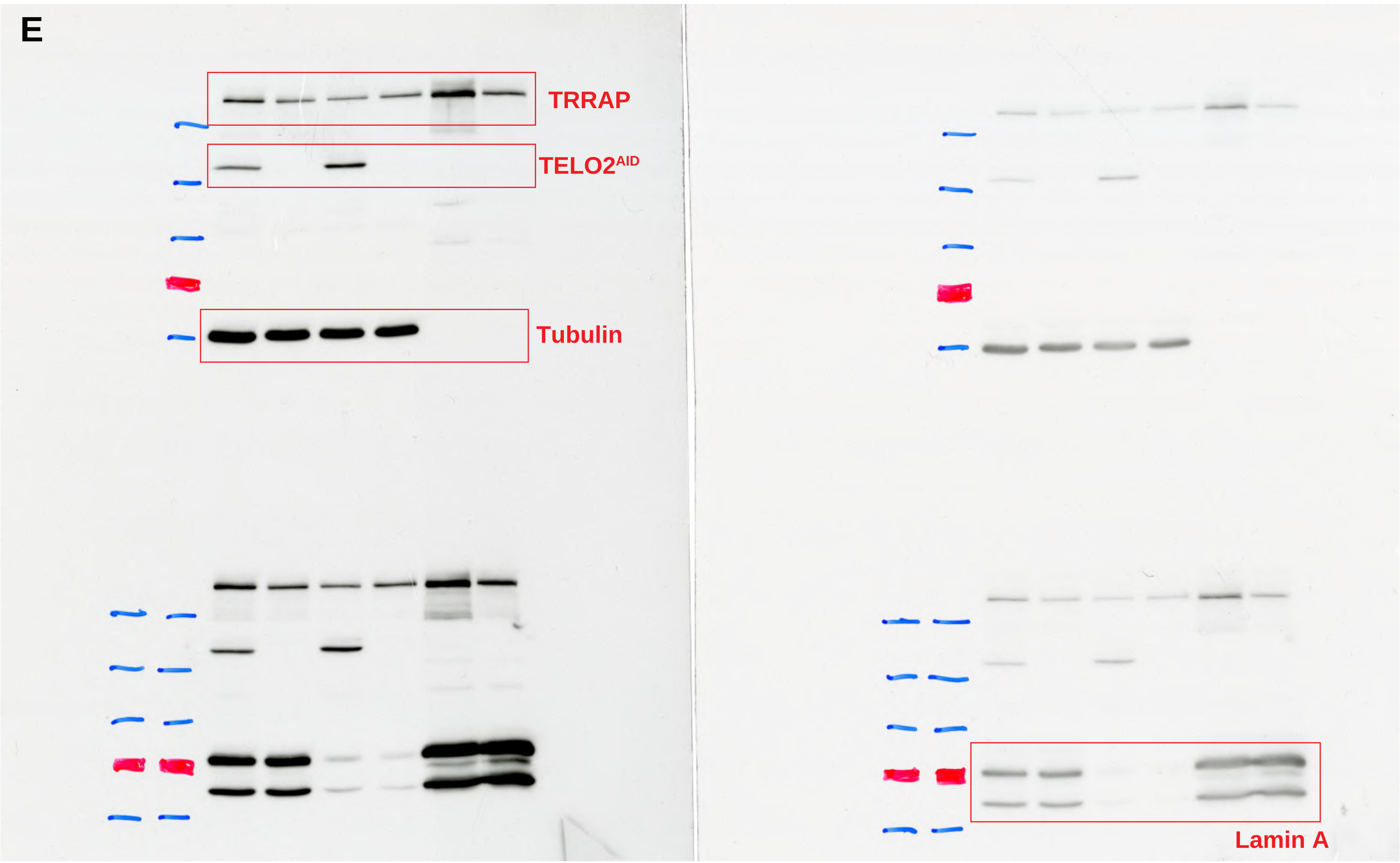
TRRAP
TELO2AID
Tubulin
Lamin A

F

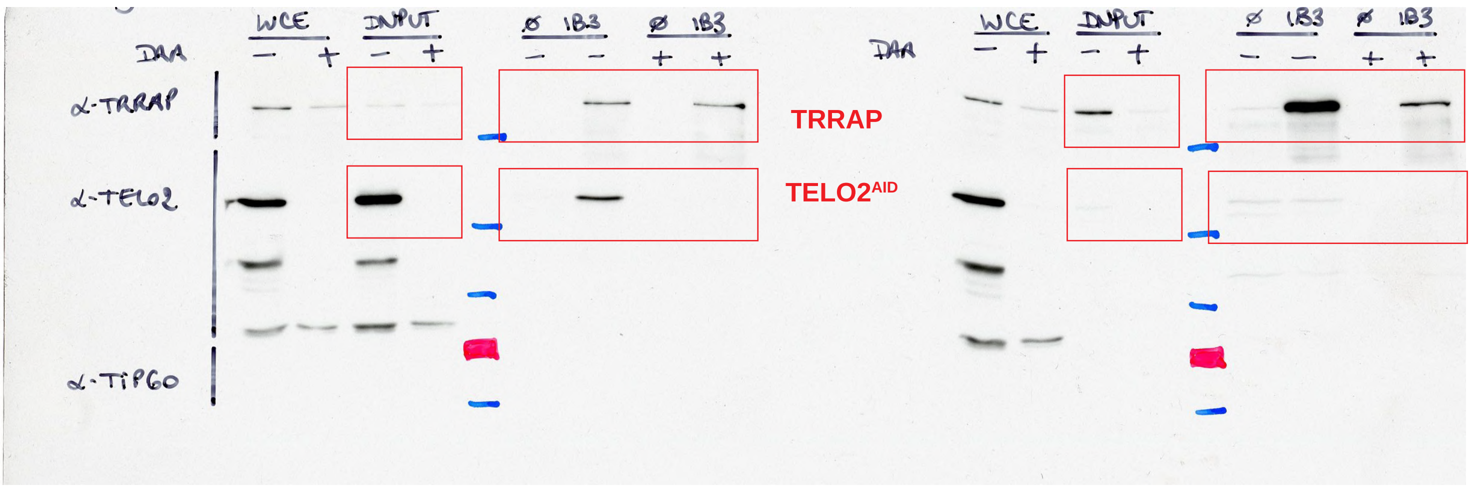
WCE
INPUT
DAA
α-TRRAP
α-TELO2
α-TIP60
TRRAP
TELO2AID
