# Supplementary material for: The TRRAP transcription cofactor represses interferon-stimulated genes in colorectal cancer cells
Source: eLife. 2022 Mar 4;11:e69705. doi: 10.7554/eLife.69705 (PMC8926402; doi:10.7554/eLife.69705)
Supplement: Transparent reporting form [file elife-69705-transrepform1.pdf]

eLIFE
1st Floor
24 Hills Road
Cambridge CB2 1JP, UK
P 01223 855340
W elifesciences.org
T @elife

eLife's transparent reporting form

We encourage authors to provide detailed information *within their submission* to facilitate the interpretation and replication of experiments. Authors can upload supporting documentation to indicate the use of appropriate reporting guidelines for health-related research (see EQUATOR Network), life science research (see the BioSharing Information Resource), or the ARRIVE guidelines for reporting work involving animal research. Where applicable, authors should refer to any relevant reporting standards documents in this form.

If you have any questions, please consult our Journal Policies and/or contact us: editorial@elifesciences.org.

Sample-size estimation

- You should state whether an appropriate sample size was computed when the study was being designed
- You should state the statistical method of sample size computation and any required assumptions
- If no explicit power analysis was used, you should describe how you decided what sample (replicate) size (number) to use

Please outline where this information can be found within the submission (e.g., sections or figure legends), or explain why this information doesn't apply to your submission:

For all experiments, the plausibility of the H0 hypothesis was estimated to be at a 1-to-1 odd ratio, meaning that we did not have any pre-conceived guess of the outcome of an experiment. Sample size was chosen based on previous experimental knowledge, which showed that n >= 3 was sufficient to detect statistically significant differences if the effect is >= 2-fold.

Replicates

- You should report how often each experiment was performed
- You should include a definition of biological versus technical replication
- The data obtained should be provided and sufficient information should be provided to indicate the number of independent biological and/or technical replicates
- If you encountered any outliers, you should describe how these were handled
- Criteria for exclusion/inclusion of data should be clearly stated
- High-throughput sequence data should be uploaded before submission, with a private link for reviewers provided (these are available from both GEO and ArrayExpress)

Please outline where this information can be found within the submission (e.g., sections or figure legends), or explain why this information doesn't apply to your submission:

eLife Sciences Publications, Ltd is a limited liability non-profit non-stock corporation incorporated in the State of Delaware, USA, with company number 5030732, and is registered in the UK with company number FC030576 and branch number BR015634 at the address 1st Floor, 24 Hills Road, Cambridge CB2 1JP | August 2014

Information about the number of replicates (*N*) can be found in each figure legend. For qualitative data, such as blot images, each legend includes a statement of how many times the experiment shown was independently replicated in the laboratory. For quantitative data, only biological replicates were considered in our analyses and for computing the standard deviation. Each graph shows the mean and the error bar, overlaid with individual data points to show the value obtained for each biological replicate.
We defined biological replicates as independent experiments performed with three independent clones isolated and purified following CRISPR-Cas9 editing.
No data were excluded from the other analyses except in case of technical failure during data acquisition. For RNAseq analyses, reads that did not align uniquely or not assigned to a feature were excluded.
RNA-seq data were uploaded to NCBI Gene Expression Omnibus and a private link for reviewers is provided.

Statistical reporting

- Statistical analysis methods should be described and justified
- Raw data should be presented in figures whenever informative to do so (typically when N per group is less than 10)
- For each experiment, you should identify the statistical tests used, exact values of N, definitions of center, methods of multiple test correction, and dispersion and precision measures (e.g., mean, median, SD, SEM, confidence intervals; and, for the major substantive results, a measure of effect size (e.g., Pearson's r, Cohen's d)
- Report exact p-values wherever possible alongside the summary statistics and 95% confidence intervals. These should be reported for all key questions and not only when the p-value is less than 0.05.

Please outline where this information can be found within the submission (e.g., sections or figure legends), or explain why this information doesn't apply to your submission:

Statistical analysis methods are described in the corresponding section of Materials & Methods. In addition, each figure legend details which test was used, which alpha treshold was decided a priori, and the number of biological replicates.
Data processing and statistical analyses were performed using Graphpad Prism (version 8.0), or R (version 3.6.0). When appropriate, we performed parametric tests (ANOVA and t-tests), therefore assuming that all our data meet the following requirements: normal distribution, equal variances, and independence of biological replicates. Normality is typically observed for continuous, relative measurements of expression. Visual examination of our data suggest that samples have similar variances. However, we did not run dedicated statistical tests, such as Shapiro-Wilk test to test for normality or the Bartlett's test for variance equality.

(For large datasets, or papers with a very large number of statistical tests, you may upload a single table file with tests, Ns, etc., with reference to sections in the manuscript.)

Group allocation

- Indicate how samples were allocated into experimental groups (in the case of clinical studies, please specify allocation to treatment method); if randomization was used, please also state if restricted randomization was applied
- Indicate if masking was used during group allocation, data collection and/or data analysis

Please outline where this information can be found within the submission (e.g., sections or figure legends), or explain why this information doesn't apply to your submission:

No randomization was required because this study was carried out using isogenic cultured cell lines grown in controlled conditions.
The investigators numbered the samples during each experiment such that data collection and analysis were performed without a priori knowledge of the genotype or culture condition.

Additional data files ("source data")

- We encourage you to upload relevant additional data files, such as numerical data that are represented as a graph in a figure, or as a summary table

- Where provided, these should be in the most useful format, and they can be uploaded as "Source data" files linked to a main figure or table
- Include model definition files including the full list of parameters used
- Include code used for data analysis (e.g., R, MatLab)
- Avoid stating that data files are "available upon request"

Please indicate the figures or tables for which source data files have been provided:

As requested, a ZIP file containing source data for all figure panels with Western blots is provided (Figure 1A-B, Figure 1E-H, Figure Supplemental 1E-F, Figure 3B-D, Figure 6B, Figure 7A-C).
Details of the scripts used for RNA-seq analyses can be found in the corresponding section of Materials & Methods. Briefly, RNA-seq data were collected on an Illumina Hiseq 4000, at Fasteris SA. Adapter sequences were trimmed from reads in the Fastq sequence files, following demultiplexing according to index barcodes. Reads were aligned using HISAT2 and counted for exon features using htseq-count. Variance-mean dependence was estimated from count tables and tested for differential expression based on a negative binomial distribution, using DESeq2.

eLife Sciences Publications, Ltd is a limited liability non-profit non-stock corporation incorporated in the State of Delaware, USA, with company number 5030732, and is registered in the UK with company number FC030576 and branch number BR015634 at the address 1st Floor, 24 Hills Road, Cambridge CB2 1JP | August 2014
